# Safety of the *Salmonella enterica* serotype Dublin strain Sdu189-derived live attenuated vaccine—A pilot study

**DOI:** 10.3389/fvets.2022.986332

**Published:** 2022-09-28

**Authors:** Fuzhong Wang, Lei Wang, Haojie Ge, Xiaobo Wang, Yaxin Guo, Zhengzhong Xu, Shizhong Geng, Xin'an Jiao, Xiang Chen

**Affiliations:** ^1^Jiangsu Key Laboratory of Zoonosis/Jiangsu Co-innovation Center for Prevention and Control of Important Animal Infectious Diseases and Zoonoses, Yangzhou University, Yangzhou, China; ^2^Key Laboratory of Prevention and Control of Biological Hazard Factors (Animal Origin) for Agrifood Safety and Quality of Ministry of Agriculture and Rural Affairs, Yangzhou University, Yangzhou, China

**Keywords:** *Salmonella enterica* serovar Dublin, *spiC*, *aroA*, live attenuated vaccine, immune protection

## Abstract

*Salmonella enterica* serovar Dublin (*S*. Dublin) is an important zoonotic pathogen with high invasiveness. In the prevention and control of the *Salmonella* epidemic, the live attenuated vaccine plays a very important role. To prevent and control the epidemic of *S*. Dublin in cattle farms, the development of more effective vaccines is necessary. In this study, we constructed two gene deletion mutants, Sdu189Δ*spiC* and Sdu189Δ*spiC*Δ*aroA*, with the parental strain *S*. Dublin Sdu189. The immunogenicity and protective efficacy were evaluated in the mice model. First, both mutant strains were much less virulent than the parental strain, as determined by the 50% lethal dose (LD_50_) for specific pathogen-free (SPF) 6-week-old female BALB/c mice. Second, the specific IgG antibody level and the expression level of cytokine TNF-α, IFN-γ, IL-4, and IL-18 were increased significantly in the vaccinated mice compared to the control group. In addition, the deletion strains were cleared rapidly from organs of immunized mice within 14 d after immunization, while the parental strain could still be detected in the spleen and liver after 21 d of infection. Compared with the parental strain infected group, no obvious lesions were detected in the liver, spleen, and cecum of the deletion strain vaccinated groups of mice. Immunization with Sdu189Δ*spiC* and Sdu189Δ*spiC*Δ*aroA* both provided 100% protection against subsequent challenges with the wild-type Sdu189 strain. These results demonstrated that these two deletion strains showed the potential as live attenuated vaccines against *S*. Dublin infection. The present study established a foundation for screening a suitable live attenuated *Salmonella* vaccine.

## Introduction

*Salmonella enterica* serovar Dublin (*S*. Dublin) is a serotype highly adapted to cattle, which causes enteritis and systemic diseases in bovine hosts (1). The clinical manifestations are watery or bloody diarrhea, accompanied by fever, depression, loss of appetite, dehydration, and bacteremia (2). It has also been reported that *S*. Dublin can cause respiratory diseases in calves of 10–30 d old, with an incidence rate of over 20% and a 75% mortality (3). *S*. Dublin is widely prevalent globally which causes enormous economic losses and severely constrains the development of the breeding industry (4). The transmission of *S*. Dublin to humans is usually associated with direct contact with infected animals or the consumption of contaminated raw meat, water, milk, and dairy products, which can lead to invasive infection and death in humans susceptible to diseases such as weakness and chronic infection (5). The consumption of beef and dried beef contaminated with *S*. Dublin has also been identified as the major cause of *S*. Dublin infection in humans (6). The traditional microbiological analysis found that *S*. Dublin has the highest isolated rate in raw milk and dairy products, meanwhile, *S*. Dublin in human blood and feces had the highest isolated rate (7).

To prevent and control the infection of this intestinal pathogen, multiple strategies need to be implemented, such as avoiding the introduction of pathogenic bacteria, controlling the transmission of pathogenic bacteria in livestock, vaccinating, and using antibiotics (8, 9). However, to avoid the introduction of pathogenic bacteria and control their transmission, farms need to spend huge human and material resources. Previous studies have also confirmed that the use of antibiotics can achieve specific therapeutic effects, which directly leads to the production of multiple drug-resistant strains and drug residues in meat (10). An American study reported that in the 8 years from 2005 to 2013 alone, the multi-drug resistance rate of *S*. Dublin increased to 55%, while the drug resistance rate of other serotypes was only 12% (11). Consequently, it will be increasingly difficult to treat *S*. Dublin infection with antibiotics, and vaccination will probably become the most important means to control *S*. Dublin infection in cattle. At present, three main types of vaccines are commercially available, including inactivated vaccine, live attenuated vaccine, and genetically engineered live attenuated vaccine (12). However, an inactivated vaccine has the disadvantages of low immunogenicity, weak protective immunity, short duration of effect, and requires multiple vaccinations (13). The drug resistance of live attenuated vaccines caused by the problem of preparation technology cannot be ignored. The genetically engineered live attenuated vaccine has shown sufficient advantages (14). In 1984, Smith and others developed a live vaccine SL1438 of the *S*. Dublin *aro*^−^ gene deletion strain, which has been approved by the U.S. Department of Agriculture (USDA), but its clinical immune efficacy and protective efficacy were still insufficient (15, 16). The *Salmonella* vaccine used in the modern cattle industry decreased morbidity and mortality, and inhibit the bacterial excretion and persistent infection of *S*. Dublin. However, the use of these vaccines did not completely prevent the spread of *S*. Dublin in livestock (17). The ideal vaccine should be able to induce both humoral and cellular immune responses, with high safety and cross-protection against other serotypes of *Salmonella* (18). Therefore, it is necessary to develop new vaccines to prevent and control the infection and transmission of *S*. Dublin in cattle.

*SpiC* is an effector protein encoded by SPI-2, which is secreted by the *Salmonella* type III secretion system (T3SS) and injected into host cells (19, 20). It has been demonstrated that deleting *spiC* gene significantly reduces the virulence of *Salmonella* in mouse and chicken models (21). The *spiC* gene deletion strain of *Salmonella* Pullorum constructed by genetic engineering has been evaluated as a candidate vaccine for *S*. Pullorum (22, 23). In the breeding process, some nutrients can be added to improve the health of animals. Based on this, we consider whether we can adjust the state of vaccine strains in the body through feeding links, to achieve a better immune effect. The *aroA* gene is part of the shikimic acid pathway, which directly links glycolysis with the synthesis of aromatic amino acids (24). Mammals cannot synthesize aromatic amino acids by themselves, so it was difficult for *S*. Dublin to reproduce in mammalian tissues or survive in the environment without the *aroA* gene (25). *aroA* gene deletion is most commonly used as a metabolic mutation to reduce the virulence of *Salmonella* and other bacteria. Previous studies have confirmed that *aroA*-deficient *S*. Dublin strain is highly attenuated and considered to be a suitable carrier system (26). Therefore, *aroA* and *spiC* were selected as the target genes for the construction of candidate vaccines.

In this study, Sdu189Δ*spiC* and Sdu189Δ*spiC*Δ*aroA* were constructed by employing the suicide plasmid pDM4. Bacterial virulence, host clearance, immune responses, pathological observation, and protective efficacy of these two deletion strains in mice model were analyzed to evaluate the potential of Sdu189Δ*spiC* and Sdu189Δ*spiC*Δ*aroA* as live attenuated vaccines against *S*. Dublin infection.

## Materials and methods

### Bacterial strains and growth conditions

Sdu189 is a clinical strain obtained in 2017 from human anal swabs in Jiangsu Province, China. The *spiC* and *aroA* gene deletion mutant strains were constructed by using the suicide vector pDM4 based on homologous recombination, as previously described (27, 28). The open reading frame (ORF) of the targeted genes was completely deleted and confirmed by PCR analysis and sequencing. All of these strains were cultured in Luria-Bertani (LB) agar medium, LB broth, and Xylose Lysine Tergitol-4 (XLT4) agar at 37°C.

### Biochemical test and growth characteristics *in vitro*

To evaluate the effect of deleted genes on the biological properties of *S*. Dublin mutants, biochemical tests were performed using the API 20E identification kit (BioMérieux, France) according to the manufacturer's protocol. The growth characteristics of Sdu189Δ*spiC*, Sdu189Δ*spiC*Δ*aroA*, and Sdu189 were determined by measuring the optical density (OD_600_) of each strain cultured in 15 ml of LB broth at 37°C with shaking at 180 rpm. The OD_600_ was monitored every hour for 10 h as previously described.

### Mouse

Specific pathogen-free (SPF) 6-week-old female BALB/c mice were obtained from Beijing Weitonglihua Laboratory Animal Technology Co., Ltd. (Beijing, China). All mice were confirmed as free from *Salmonella* infection by both bacteriological examination and serum detection. Each group of mice was raised in separate rearing isolators and supplied with commercial feed and drinking water. The food and water for mice were tested to be *Salmonella* negative. All of the animal experiments and management procedures were undertaken with the permission of the Animal Welfare and Ethics Committees of Yangzhou University (IACUC license number: YZUDWLL-201811-001) and complied with the guidelines of the institutional administrative committee and the ethics committee for laboratory animals.

### Virulence assessment

To evaluate the virulence of Sdu189Δ*spiC* and Sdu189Δ*spiC*Δ*aroA*, 96 mice were divided into 16 groups (*n* = 6) randomly. Mice were administered 100 μl of Sdu189Δ*spiC*, Sdu189Δ*spiC*Δ*aroA* (1 × 10^10^, 1 × 10^9^, 1 × 10^8^, 1 × 10^7^, or 1 × 10^6^ CFU/ml), or Sdu189 (1 × 10^7^, 1 × 10^6^, 1 × 10^5^, 1 × 10^4^, or 1 × 10^3^ CFU/ml) in phosphate-buffered saline (PBS) by intramuscular injection. Control mice received 100 μl of PBS in the same way. The clinical symptoms and death of mice were recorded every day within 3 weeks after infection, and the LD_50_ was calculated by the Reed-Muench method (29).

### Persistence and clearance of bacteria in mice tissues and organs

A total of 63 6-week-old mice were randomly divided into three groups (*n* = 21). Each group was immunized intramuscularly with 1 × 10^5^ CFU of Sdu189, Sdu189Δ*spiC*, or Sdu189Δ*spiC*Δ*aroA*. Sections of the liver, spleen, ileum, and cecum were aseptically collected at 1, 3, 5, 7, 9, 14, and 21 d post-immunization. The organs were weighed and suspended in 1 ml of PBS for homogenization. The homogenates were diluted serially and subsequently inoculated on XLT4 agar plates for bacterial recovery at 37°C. After overnight cultivation, the bacterial number was counted and reported as log_10_ CFU/g.

### Immune response

The humoral immune response was evaluated by measuring specific IgG titers by indirect enzyme-linked immunosorbent assay (ELISA), using the heat-killed Sdu189 strain as the coating antigen. Mice blood samples were collected at 7, 14, and 21 d post-immunization, stored at 4°C for 4 h, and then centrifuged at 3,500 rpm for 10 min. Serum samples were diluted continuously as primary antibodies. Horseradish peroxidase (HRP)-conjugated goat anti-mouse IgG (1:10,000 dilution, Sigma-Aldrich) was used as the secondary antibody. The HRP activity was determined using 3,30,5,50-tetramethylbenzidine (TMB, Sigma-Aldrich), and the OD_450_ value was determined with an ELISA reader (BioTek, USA) to detect the level of humoral immunity.

To evaluate the T-cell immune response and inflammatory response induced by the vaccine strains, the mRNA expression of cytokines IFN-γ, TNF-α, IL-4, and IL-18 was measured by qRT-PCR analysis with the Synthetic dye method (30, 31). GAPDH was used as an endogenous control gene. Gene-specific primers used in this analysis were listed in Table 1. Total RNA was extracted from spleen samples collected at 7, 14, and 21 d post-vaccination by using an RNA Plus Mini kit (Qiagen, Germany) according to the manufacturer's instructions. The RNA was reverse transcribed into cDNA by using PrimeScript RT Reagent Kit (TaKaRa) according to the manufacturer's instructions and then subjected to qRT-PCR analysis. The comparative threshold cycle (2–^ΔΔ*C*(*T*)^ method) was used to calculate relative concentrations. All qRT-PCR reactions were performed in triplicates and repeated three times.

**Table 1:** Primers used for qRT-PCR of mouse cytokines.

**Gene amplified**	**prrimers**	**Primers sequences (5^′^-3^′^)**
IFN-γ	IFN-γ-F	AGCAACAACATAAGCGTCAT
	IFN-γ-R	CTCAAACTTGGCAATACTC
TNF-α	TNF-α-F	TCTCATTCCTGCTTGTGG
	TNF-α-R	ACTTGGTGGTTTGCTACGA
IL-4	IL-4-F	TCACAGCAACGAAGAACACC
	IL-4-R	CGAAAAGCCCGAAAGAGTC
IL-10	IL-10-F	ACCTGGTAGAAGTGATGCC
	IL-10-R	GACACCTTGGTCTTGGAG
IL-18	IL-18-F	AAGAGGACTGGCTGTGACC
	IL-18-R	TTGGCAAGCAAGAAAGTGTC
GAPDH	GAPDH-F	CAAATTCAACGGCACAGTCA
	GAPDH-R	TTAGTGGGGTCTCGCTCC

### Immune protection assessment

To evaluate the protective efficacy of these two vaccine candidates, 36 6-week-old mice were divided into three groups (*n* = 12) randomly. Mice were immunized intramuscularly with 1 × 10^7^ CFU of Sdu189Δ*spiC* or Sdu189Δ*spiC*Δ*aroA* in 100 μl of PBS, and the control mice received 100 μl PBS. Two weeks post-infection, mice were immunized again with the same mutants using the same route and dose. Two weeks after the secondary immunization, each group of mice was challenged intramuscularly with 2 × 10^8^ CFU of Sdu189. The number of surviving mice was recorded, and clinical symptoms were observed daily for 3 weeks. Samples of the liver, spleen, and cecum of each group were collected and fixed in 10% neutral-buffered formalin at 1, 3, 5, 7, 14, and 21 d post-immunization. The fixed samples were dehydrated, embedded in paraffin wax, sectioned to 5 μm, and stained with hematoxylin and eosin.

### Statistical analysis

All data were described as mean ± standard error of the mean (SEM) unless otherwise specified and were analyzed using GraphPad Prism version 7.0. A *p*-value of <0.05 was considered statistically significant.

## Results

### Biochemical characteristics and growth characteristics of gene deletion mutants

**Figure 1:**
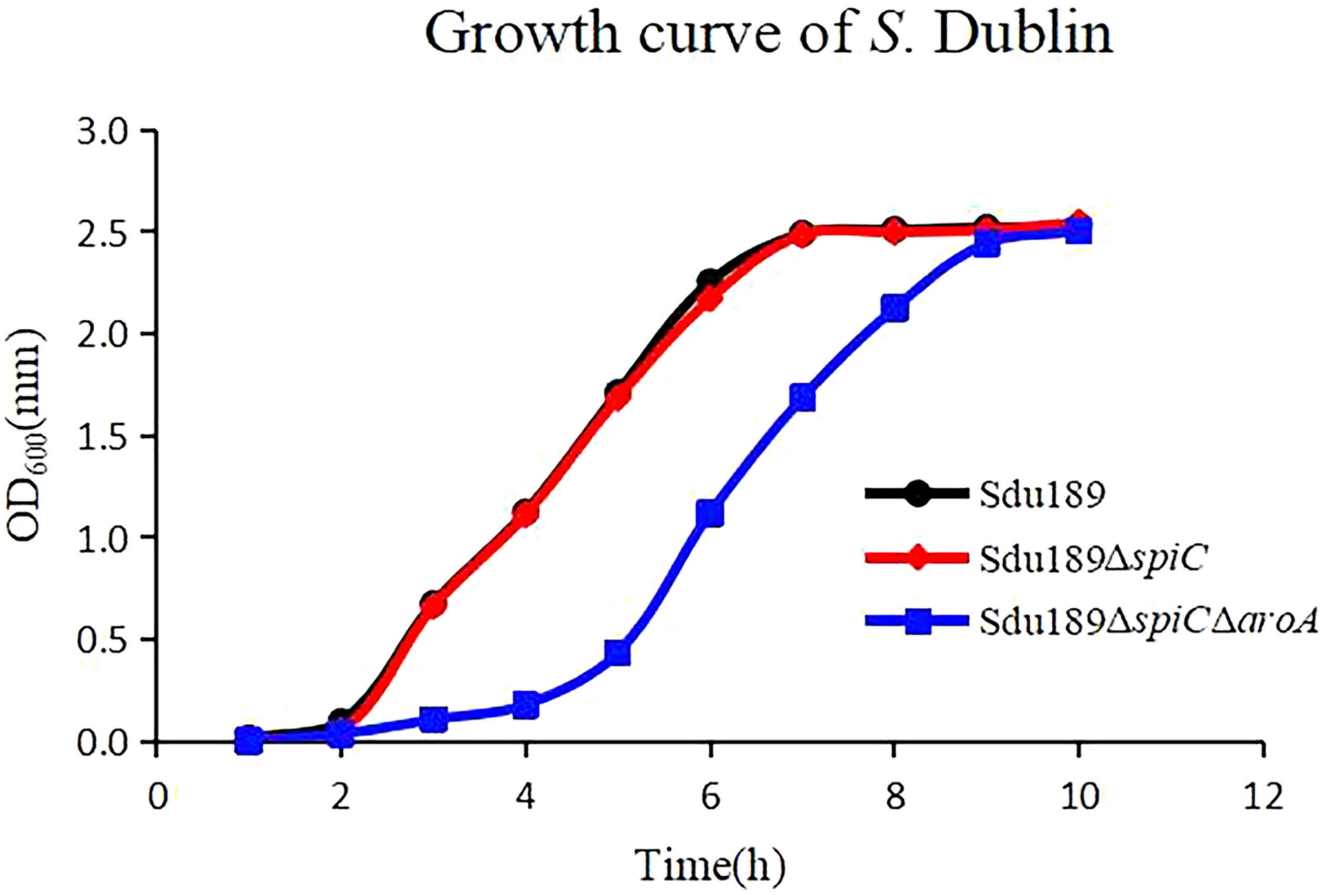
Growth curves of Sdu189, Sdu189Δ*spiC*, and Sdu189Δ*spiC*Δ*aroA*. Bacteria were grown in LB broth at 37°C for 10 h with agitation, and the OD_600_ values of triplicate cultures in LB medium were determined in 1-h intervals.

The API 20E identification kit was used to test the physiological and biochemical characteristics of deletion strains and parental strains. The results showed that the deletion of *spiC* and *aroA* genes markedly influenced bacterial metabolism (unpublished), and the knockout of the *aroA* gene in Sdu189 inhibited the ability to produce H_2_S. In addition, the growth curve of Sdu189Δ*spiC* was similar to the parental strain, reaching the logarithmic growth period at about 2 h, and gradually entering the stable period after 7 h (Figure 1). However, as we predicted, the growth curve of Sdu189Δ*spiC*Δ*aroA* was different, reaching the logarithmic growth period at about 5 h, and gradually entering the stable period after 9 h.

### Deletion of *spiC* and *aroA* led to reduced virulence of Sdu189 in mice model

**Figure 2:**
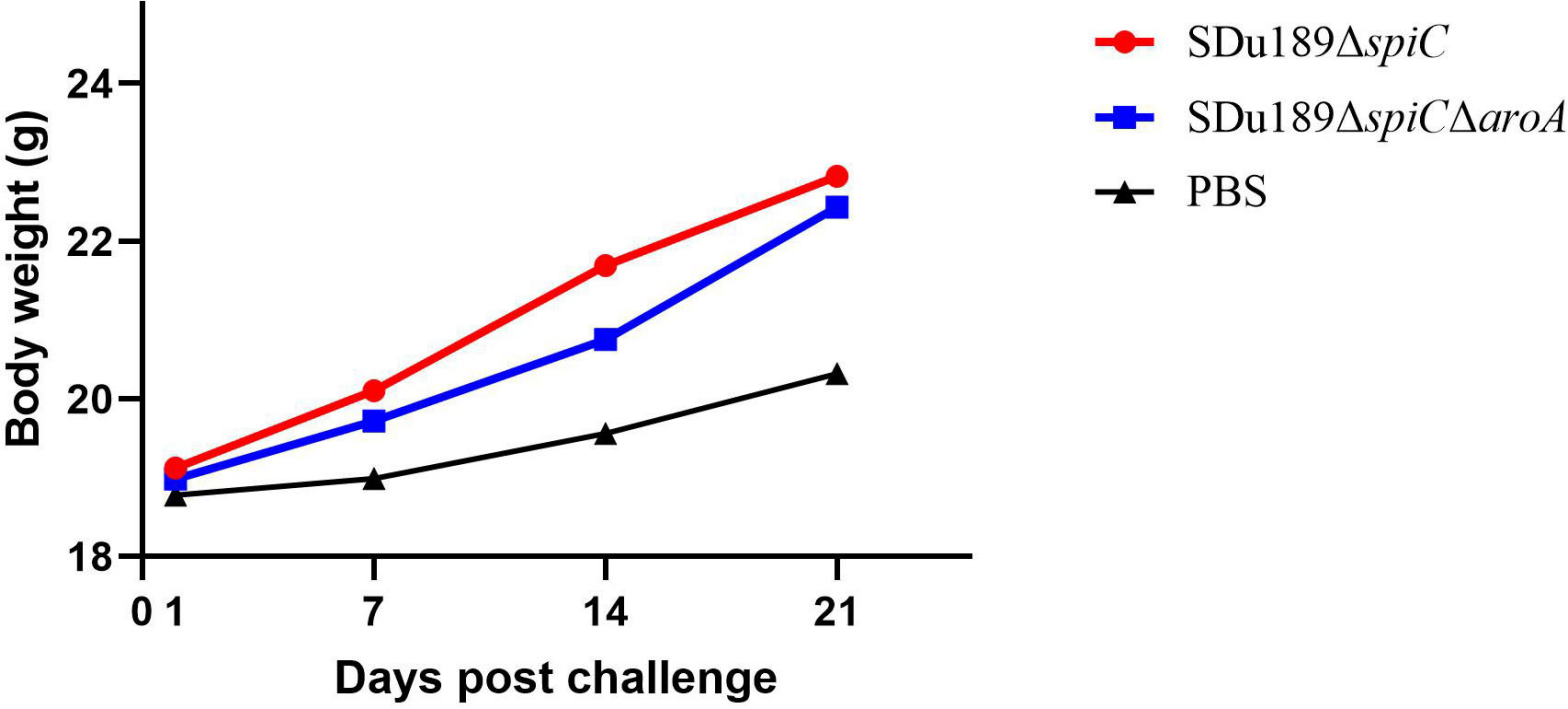
The body weight of mice after immunization. Groups of 6-week-old BALB/c mice were intramuscularly immunized with Sdu189Δ*spiC* and Sdu189Δ*spiC*Δ*aroA*, and the control group received 100 μl PBS. The body weights of the experimental mice were determined at 1, 7, 14, and 21 days post-immunization. Data are presented as mean ± SEM.

The virulence of Sdu189, Sdu189Δ*spiC*, and Sdu189Δ*spiC*Δ*aroA* was evaluated in 6-week-old BALB/c mice with intramuscular injection. As shown in Table 2, the LD_50_ of Sdu189Δ*spiC* and Sdu189Δ*spiC*Δ*aroA* strains were 586-fold and 1,073-fold higher than that of parental strain Sdu189, respectively. In addition, there was no significant difference in the weight of decimals between the two groups compared with the PBS group (Figure 2).

**Table 2:** The LD_50_ of *S*. Dublin Sdu189, Sdu189Δ*spiC* and Sdu189Δ*spiC*Δ*aroA*, in 6-week-old mice after intramuscular immunization.

**Strains**	**Challenge dose (CFU)**	**No. of deaths/total No. of mice**	**LD_50_ (CFU)**
Sdu189	1.2 × 10^7^	6/6	
	1.2 × 10^6^	4/6	
	1.2 × 10^5^	2/6	3.79 × 10^5^
	1.2 × 10^4^	0/6	
	1.2 × 10^3^	0/6	
Sdu189Δ*spiC*	1.5 × 10^10^	6/6	
	1.5 × 10^9^	6/6	
	1.5 × 10^8^	2/6	2.22 × 10^8^
	1.5 × 10^7^	0/6	
	1.5 × 10^6^	0/6	
Sdu189Δ*spiC*Δ*aroA*	1.26 × 10^10^	6/6	
	1.26 × 10^9^	5/6	
	1.26 × 10^8^	1/6	4.07 × 10^8^
	1.26 × 10^7^	0/6	
	1.26 × 10^6^	0/6	

### Colonization and persistence of bacteria in tissues and organs of mice

**Figure 3:**
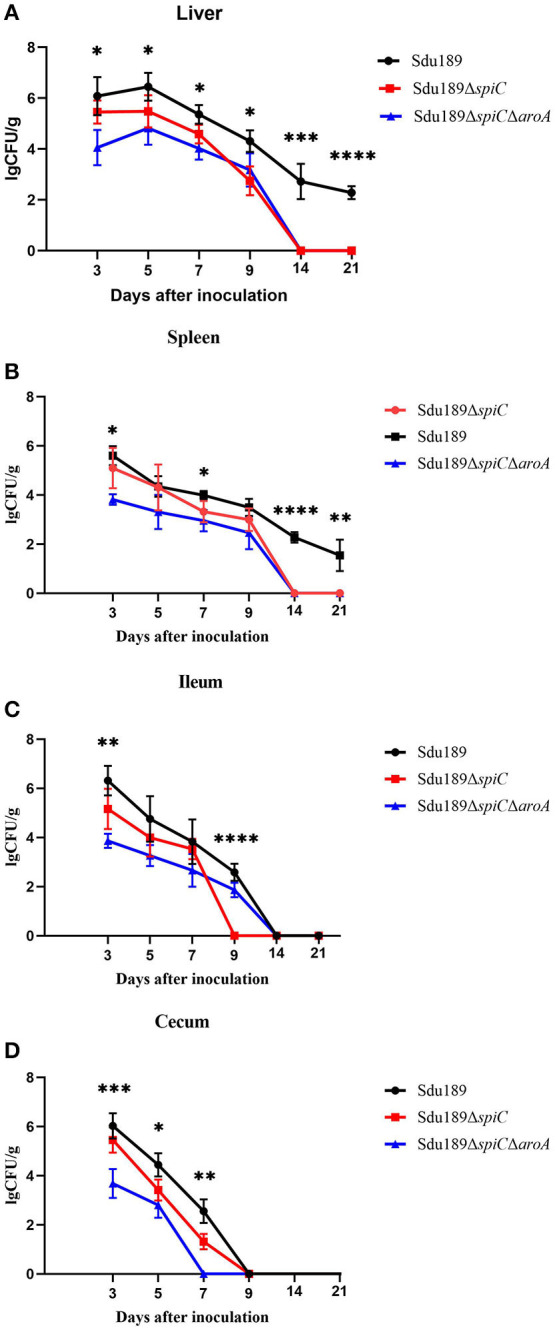
Bacterial colonization in tissues and organs of immunized mice. Mice were immunized with parental strain Sdu189, vaccine candidates Sdu189Δ*spiC* and Sdu189Δ*spiC*Δ*aroA*. Bacterial colonization in the liver **(A)**, spleen **(B)**, ileum **(C)**, and cecum **(D)** of the immunized mice. *p < 0.05, **p < 0.01, ***p < 0.001, and ****p < 0.0001 compared with the bacterial colonization number of control group mice by one-way ANOVA followed by Bonferroni's multiple comparison test. Data are presented as mean ± SEM of log10 CFU/g.

The bacterial number was calculated in the liver, spleen, ileum, and cecum of immunized mice. All tissue samples from the negative control group were negative for *S*. Dublin. As shown in Figure 3, Sdu189, Sdu189Δ*spiC*, and Sdu189Δ*spiC*Δ*aroA* colonization reached the highest level at 1 d post-immunization, thereafter it decreased gradually in the cecum. The bacterial colonization reached the highest level at 3 d post-immunization in the spleen and ileum, while the peak colonization of *S*. Dublin was found at 5 d post-immunization in the liver.

The viable counts in the liver, spleen, ileum, and cecum from the Sdu189Δ*spiC*Δ*aroA* immunization group were lower than those from mice inoculated with Sdu189 at all tested time points. Overall, the bacterial clearance in Sdu189Δ*spiC* immunization group was also enhanced compared with the Sdu189 immunization group. None of *S*. Dublin was detected in the spleen and liver from mice immunized with Sdu189Δ*spiC* or Sdu189Δ*spiC*Δ*aroA* at 14 d post-immunization. While, spleen and liver samples from mice immunized with Sdu189 were still positive at 21 d post-immunization (Figures 3A,B). In addition, all ileum samples from Sdu189Δ*spiC* immunization group were negative for bacterial recovery at 9 d post-immunization. And none of *S*. Dublin was detected in cecum samples from Sdu189Δ*spiC*Δ*aroA* immunization group at 5 d post-immunization. These results indicated that the colonization ability of both Sdu189Δ*spiC* and Sdu189Δ*spiC*Δ*aroA* was decreased compared with wild strain Sdu189.

### Analysis of histopathological changes or lesions of mouse organs

Subsequently, we analyzed the histopathological changes or lesions of the organs from mice infected with *S*. Dublin. The organs from the Sdu189 infected group of mice showed obvious splenic sinus expanding, the number of macrophages increasing, and infiltration of heterophilic granulocytes at 3 d post-challenge (Supplementary Figure S1). Furthermore, the spleen and liver from the mice of the Sdu189 infection group displayed severe focal necrosis, and massive macrophage infiltration at 7–21 d post-challenge (Supplementary Figure S2). No obvious histopathological changes were found in the liver, spleen, and cecum among mice originating from immunization groups. These data suggested that the vaccine candidate strains showed a great safety profile in the host.

### Humoral and cellular immune response after immunization

**Figure 4:**
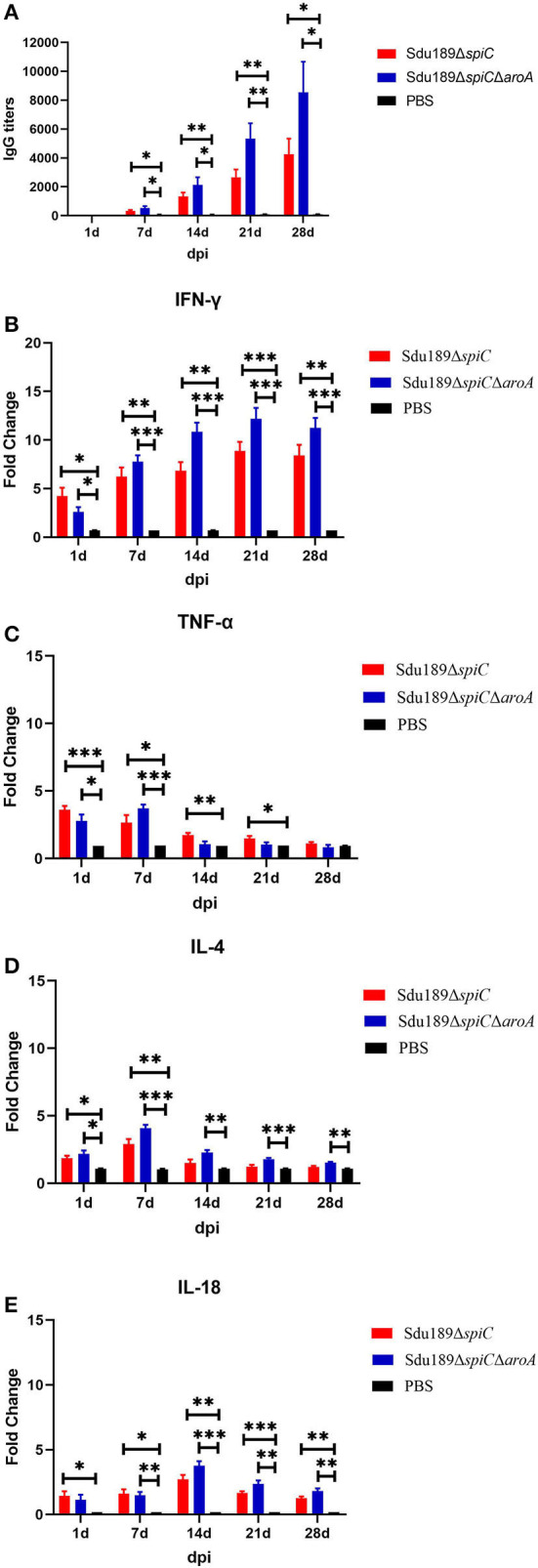
Humoral and cellular immune responses induced by vaccine candidates. The 6-week-old mice were immunized with 1×10^5^ CFU of Sdu189Δ*spiC* or Sdu189Δ*spiC*Δ*aroA*, and the serum *S*. Dublin-specific IgG antibody titer at 1, 7, 14, 21, and 28 d post-immunization were detected by Elisa **(A)**. The total RNA was extracted from spleens of mice vaccinated by mutants at 1, 7, 14, 21, and 28 d post-immunization and subsequently subjected to the qRT-PCR analysis for detecting the expression levels of IFN-γ **(B)**, TNF-α **(C)**, IL-4 **(D)**, and IL-18 **(E)**. *p < 0.05, **p < 0.01, and ***p < 0.001 compared with control group by one-way ANOVA followed by Bonferroni's multiple comparison test. Data are presented as mean ± SEM.

To evaluate the humoral immune response induced by two deletion strains, the serum IgG level was measured by indirect ELISA. As compared to the control group, the serum *S*. Dublin antigen-specific IgG levels were significantly increased in Sdu189Δ*spiC* and Sdu189Δ*spiC*Δ*aroA* immunization group at 14, 21, and 28 d post-immunization. The serum IgG levels of Sdu189Δ*spiC*Δ*aroA* immunized group of mice were significantly higher than those of mice immunized with Sdu189Δ*spiC* (Figure 4A). These results demonstrated that both Sdu189Δ*spiC* and Sdu189Δ*spiC*Δ*aroA* were able to strongly elicit humoral immune responses in mice, and the specific serum IgG level induced by Sdu189Δ*spiC*Δ*aroA* was stronger. To determine the expression of cytokines mRNA in the spleen of immunized mice, qRT-PCR analysis was performed using GAPDH as the internal control. As shown in Figure 4, the mRNA levels of IFN-γ and IL-18 in the spleen of both Sdu189Δ*spiC* and Sdu189Δ*spiC*Δ*aroA* immunized mice were strongly induced at 1 d post-immunization and remained high at 28 d post-immunization compared with that in the spleen of control mice. And the expression level of IFN-γ induced by Sdu189Δ*spiC*Δ*aroA* was higher than Sdu189Δ*spiC* at 14, 21, and 28 d post-immunization. Sdu189Δ*spiC*Δ*aroA* induced a remarkably higher level of IL-18 expression in mice spleen compared with Sdu189Δ*spiC*.

The mRNA expression level of TNF-α as well as that of IL-4 was significantly upregulated in the spleen of mice at 1 d after immunization with Sdu189Δ*spiC* or Sdu189Δ*spiC*Δ*aroA*, (Figure 4D) peaked at 7 d post-immunization and declined to basal level at 21 d post-immunization. These results suggested that Sdu189Δ*spiC* and Sdu189Δ*spiC*Δ*aroA* tended to induce a Th2 immune response in mice at the early stage of immunization, while these two mutants tended to induce a Th1 immune response in mice after 7 d post-immunization. On balance, immunization with Sdu189Δ*spiC* or Sdu189Δ*spiC*Δ*aroA* could induce strong cellular immune responses in the mice model.

### Immune protection

**Figure 5:**
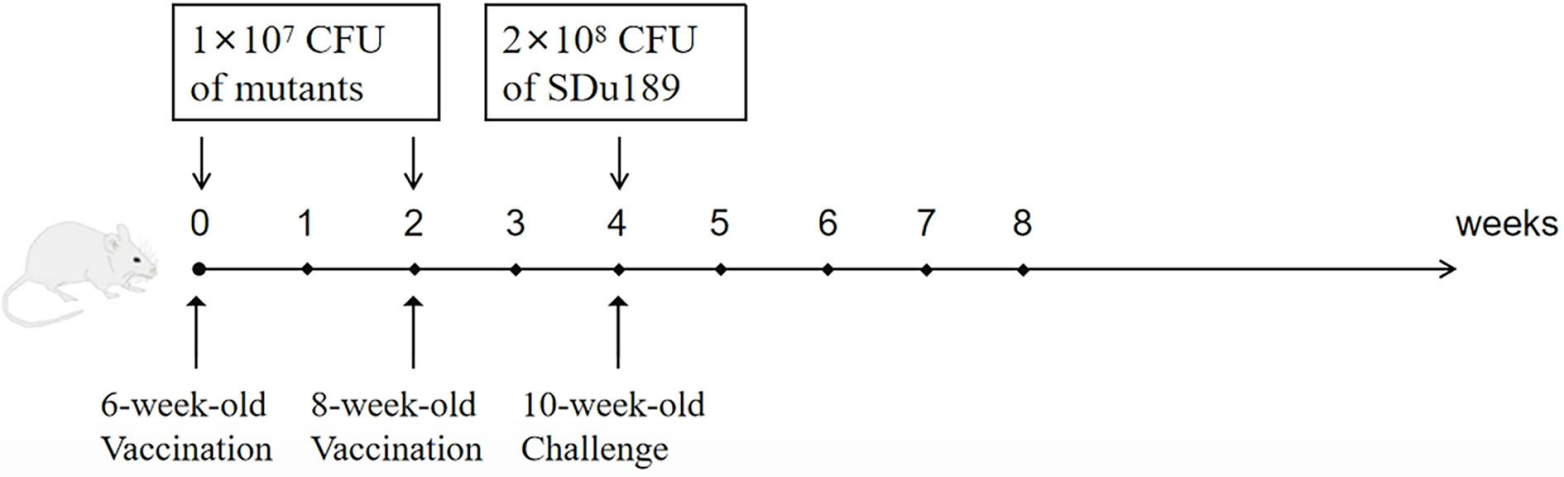
BALB/c mouse immunization protocol and protective efficacy of Sdu189 Δ*spiC* or Sdu189Δ*spiC*Δ*aroA*. Mice immunized with different doses of Sdu189 Δ*spiC*, Sdu189Δ*spiC*Δ*aroA*, or PBS were intramuscularly challenged at 2 weeks post-immunization with virulent *S*. Dublin strain Sdu189, and mortality was recorded.

**Figure 6:**
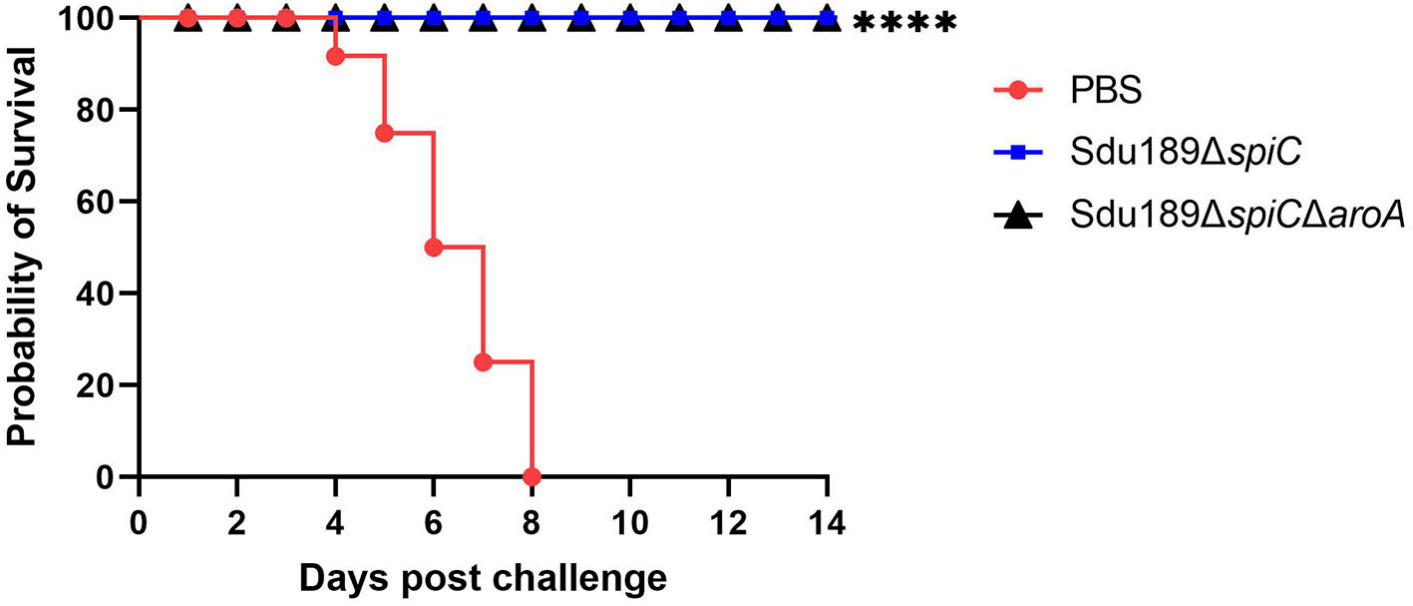
Protective efficiency of two vaccine candidates in balb/c mouse. * *p* < 0.05, ** *p* < 0.01, *** *p* < 0.001 and **** *p* < 0.0001 compared with the survival number of mice of control group mice by one-way ANOVA followed by Bonferroni's multiple comparison test.

The mice vaccinated intramuscularly with Sdu189Δ*spiC* or Sdu189Δ*spiC*Δ*aroA* were challenged with the parental strain Sdu189 at 14 d post-secondary immunization (Figure 5). The percentage of surviving mice at 14 d post-challenge was shown in Figure 6. None of the immunized mice died in Sdu189Δ*spiC* and Sdu189Δ*spiC*Δ*aroA* vaccinated groups. Whereas, all 12 mice in the control group died within 14 d post-challenge. The Sdu189Δ*spiC* and Sdu189Δ*spiC*Δ*aroA* both conferred 100% protection against a lethal *S*. Dublin challenge in mice after second intramuscular injections. Whereas, 100% mortality was observed in the control group. Furthermore, vaccinated mice did not show clinical symptoms after the challenge. Compared with the non-immunized group, the increase in body weight of the mice immunized with the deletion strain was higher than that of the non-immunized group (Figure 2).

## Discussion

*S*. Dublin is a strongly adapted serotype *Salmonella* in cattle, with high invasiveness and usually causes serious clinical symptoms and high mortality (32, 33). At present, the epidemiological investigation of *S*. Dublin has not been able to explore an effective and reliable control scheme. More research is needed to prevent the infection and transmission of *S*. Dublin in cattle farms. Vaccination is considered the best long-term way to control salmonellosis. Until now, two main types of vaccines have been developed, namely inactivated vaccine and live attenuated vaccine, for the control of *S*. Dublin in the cattle industry (34). Inactivated vaccines can induce the production of specific IgG antibodies to kill extracellular bacteria, but these antibodies are difficult to eliminate intracellular *S*. Dublin, which can be achieved by the live attenuated *Salmonella* vaccine (35, 36). Live attenuated *Salmonella* vaccine should be attenuated and safe to the host, with satisfied immunogenicity and great immune protection efficacy (37). The protective effect of the live attenuated vaccine was higher than that of an inactivated vaccine, the major reason was that the immune response induced by the live vaccine was stronger than inactivated vaccine (38, 39). In this study, *spiC* deletion strain and *spiC*/*aroA* double gene deletion strain were constructed based on the clinically isolated *S*. Dublin strain Sdu189. As we expected, the growth of Sdu189Δ*spiC*Δ*aroA* was inhibited, and it would still reach a stable growth period similar to the parent plant in the later stage (Figure 1). It shows that when Sdu189Δ*spiC*Δ*aroA* was used as a vaccine strain, some measures could be explored in the feeding link to regulate the state of bacteria. As shown in Table 2, the virulence of these two mutant strains was significantly attenuated compared with the parental strain, which meets the requirements of reducing bacterial virulence in the development of live attenuated vaccines. In addition, the double deletion mutant strains showed greater decreases in virulence, suggesting that Sdu189Δ*spiC*Δ*aroA* was safer than Sdu189Δ*spiC*.

The primary importance for the host immunized with live vaccines is safety. Previous studies showed that abscess of the inoculation site and transient diarrhea may be induced by some live *Salmonella* vaccines (40). In contrast, the immunization with Sdu189Δ*spiC* or Sdu189Δ*spiC*Δ*aroA* did not affect the body weight of mice, and no obvious clinical signs were found in immunized mice. A previous study showed that the immunization with a *nuoG* gene deletion *Salmonella* Gallinarum strain significantly reduce the mortality of chicken, whereas necrotic lesions were detected in the spleen and liver of immunized animals (41). Another live *Salmonella* Enteritidis vaccine was also reported to induce pathological changes in the tissues of the immunized chickens (42). Notably, no obvious pathological lesions were found in the spleen, liver, cecum, and ileum of mice immunized with Sdu189Δ*spiC* or Sdu189Δ*spiC*Δ*aroA*, indicating that both single deletion and double deletion live vaccines strains were safe. In addition, vaccine strain persistence and clearance in the immunized host was also an important index to evaluate the safety of vaccines. Residual vaccine strains in immunized animals may result in the contamination of slaughterhouses and meat. A live *Salmonella* vaccine strain was able to preserve in the organs of immunized animals for a long time, which led to systemic disease (43). Chickens were still positive for *Salmonella* in cloacal swabs at 10 weeks post-immunization with another live *Salmonella* vaccine (44). While nine kinds of *Salmonella* vaccines were reported to be removed quickly from the spleen of immunized animals within 14 d after immunization (45). Similar results were found in this study, both Sdu189Δ*spiC* and Sdu189Δ*spiC*Δ*aroA* can be cleared rapidly from organs of immunized mice with 14 d post-inoculation. It is undeniable that the clearance rate of vaccinated animals is an important aspect of vaccine efficacy evaluation. In addition, images of animal organs (including gross and histology) attacked by parental strains are also strong evidence for the protective efficacy of vaccines. As a preliminary inquiry, this study lacks the above evaluation data. It is necessary to use younger mice and calves for evaluation in the next evaluation to improve the value of the vaccine.

After *Salmonella* infection, the host humoral immunity and cellular immunity are indispensable in the process of eliminating pathogens. One field experiment showed that the low morbidity of salmonellosis in vaccinated flocks was largely due to the strong IgG antibody production (23). The *S*. Pullorum *spiC* mutant strain had been demonstrated to induce high levels of IgG antibody in chickens inoculated intramuscularly (46). Similarly, the *S*. Dublin-specific serum IgG levels in mice immunized with Sdu189Δ*spiC* or Sdu189Δ*spiC*Δ*aroA* were significantly higher than those of the control mice (*p* < 0.01). The early humoral immune response induced by *S*. Dublin infection was usually not enough to effectively eliminate pathogens. The cellular immune responses also play an essential role in immune defense against S. Dublin infection (47). Intracellular *Salmonella* inhibits cell division and toxicity to escape the host defense mechanism and persists in the host cell. In the acute infection stage of *S*. Dublin, the host Th1 and Th2 immune responses were quickly induced to repress the replication of intracellular bacteria and subsequently remove these invading bacteria (48). However, in the second stage of persistent infection, the reduction of Th1 type response destroys the balance between Th1 and Th2. A new balance between host and persistent *Salmonella* infection has been established (11). Previous studies showed that elimination of primary *Salmonella* infection was associated with secretion of IFN-γ (49). High IFN-γ expression mediated by Th1 immune response was reported to be essential for clearance of *Salmonella* (50), which may explain why Sdu189Δ*spiC* and Sdu189Δ*spiC*Δ*aroA* were cleared rapidly in organs of immunized mice. All of the mice immunized with these two deletion strains represented high expression levels of IFN-γ in the spleen at all tested time points after immunization, which was consistent with the findings that the live *Salmonella* vaccine was able to induce a Th1 immune response after immunization (51).

Immune protective efficacy is the most important indicator to evaluate the potential of attenuated strain as a live vaccine against pathogens infection. The live vaccines were known to have a higher protective effect than the killed vaccines. A previous study showed that a live strain only offers 80% protection in immunized animals against *S*. Enteritidis infection (52). While a live *spiC* and *crp* deletion mutant of *S*. Gallinarum vaccine strain provides 100% protection in chickens after challenge (53). Similar results were found in this study, Sdu189Δ*spiC* and Sdu189Δ*spiC*Δ*aroA* both conferred 100% protection against a lethal *S*. Dublin challenge in mice, and no obvious lesions were found in all of the tested organs of immunized mice. These data indicated the potential of Sdu189Δ*spiC* and Sdu189Δ*spiC*Δ*aroA* as effective vaccines against *S*. Dublin infection. We know that *S*. Dublin is more pathogenic to calves. The 6-week-old mice used in this experiment are not representative of calves. For further evaluation, animals younger than the 6-week-old mice need to be selected for immunization to make up for this deficiency.

In summary, the live attenuated *S*. Dublin strains Sdu189Δ*spiC* and Sdu189Δ*spiC*Δ*aroA* were able to induce both strong humoral and cellular immune responses in mice, accompanied by high safety. These two vaccine strains were also conferred high protection against the lethal *S*. Dublin challenge in mice. Thus, the Sdu189Δ*spiC* and Sdu189Δ*spiC*Δ*aroA* strains have the potential of being a safe, immunogenic, and effective vaccine against *S*. Dublin infection.

## Data availability statement

The original contributions presented in the study are included in the article/Supplementary material, further inquiries can be directed to the corresponding author/s.

## Ethics statement

The animal study was reviewed and approved by Animal Welfare and Ethics Committees of Yangzhou University.

## Author contributions

FW and LW contributed to the conception and design of the study. HG organized the database. FW and XW performed the statistical analysis. FW wrote the first draft of the manuscript. YG, ZX, SG, and XC wrote sections of the manuscript. All authors contributed to manuscript revision, read, and approved the submitted version.

## Funding

This work received financial support from the National Key Research and Development Program of China (2021YFD1800403), the Jiangsu Agricultural Science and Technology Independent Innovation Funds (CX(21)1004), the Science and Technology Program of Jiangsu (BE2021331), and the Priority Academic Program Development of Jiangsu Higher Education Institutions (PAPD).

## Conflict of interest

The authors declare that the research was conducted in the absence of any commercial or financial relationships that could be construed as a potential conflict of interest.

## Publisher's note

All claims expressed in this article are solely those of the authors and do not necessarily represent those of their affiliated organizations, or those of the publisher, the editors and the reviewers. Any product that may be evaluated in this article, or claim that may be made by its manufacturer, is not guaranteed or endorsed by the publisher.
