# Supplementary material for: Safety of the Salmonella enterica serotype Dublin strain Sdu189-derived live attenuated vaccine—A pilot study
Source: Front Vet Sci. 2022 Sep 28;9:986332. doi: 10.3389/fvets.2022.986332 (PMC9554587; doi:10.3389/fvets.2022.986332)
Supplement: Supplementary file 1 [file Presentation_1.pdf]

# *Supplementary Material*

## 1 Supplementary Figures

(A)
Liver

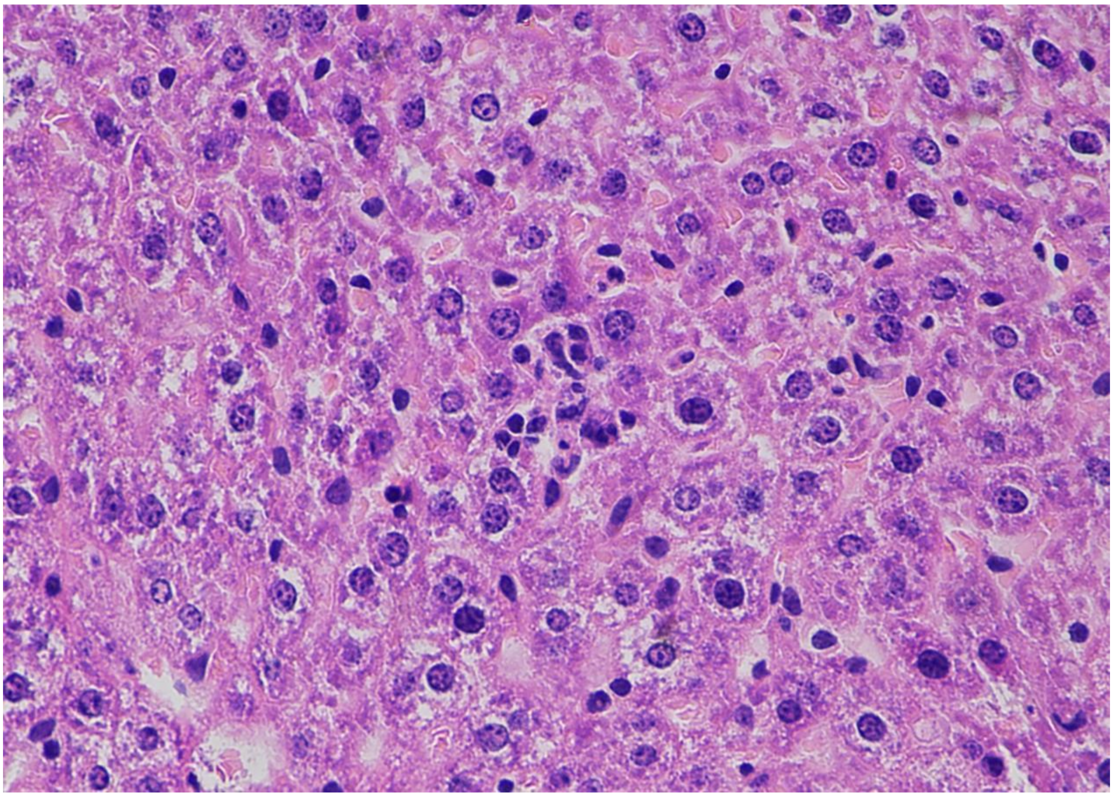

(B)
Spleen

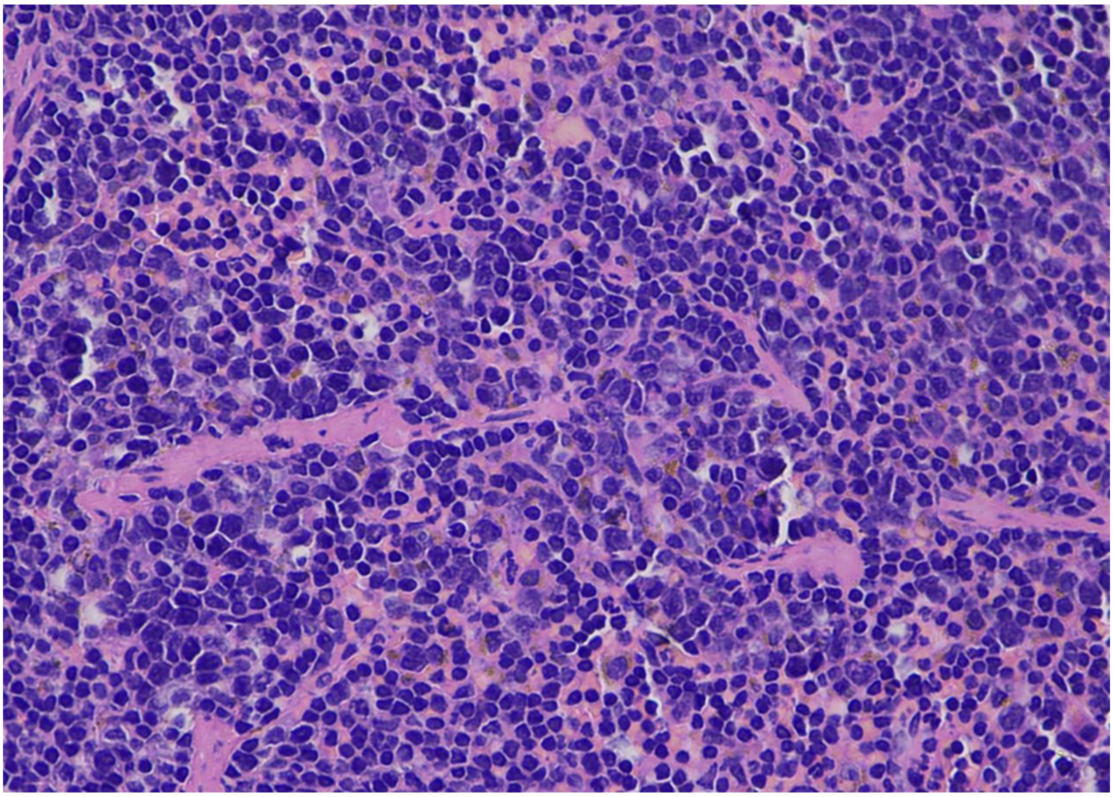

(C)
Cecum

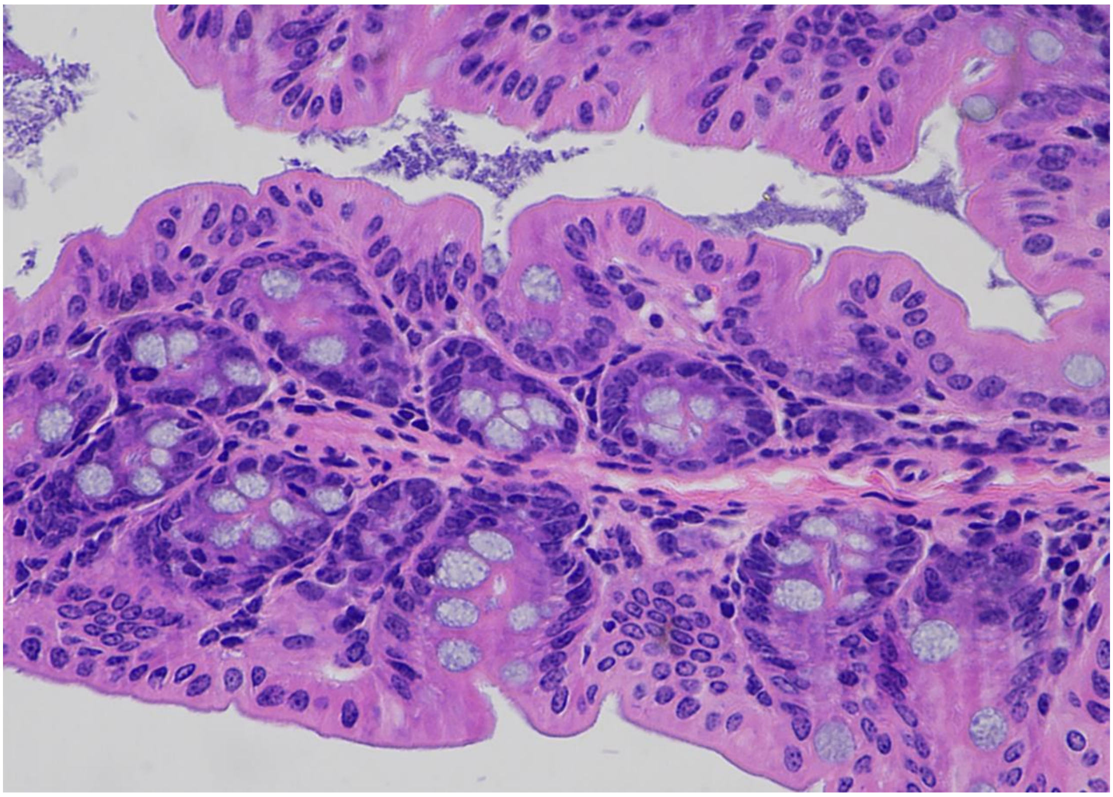

(D)
Liver

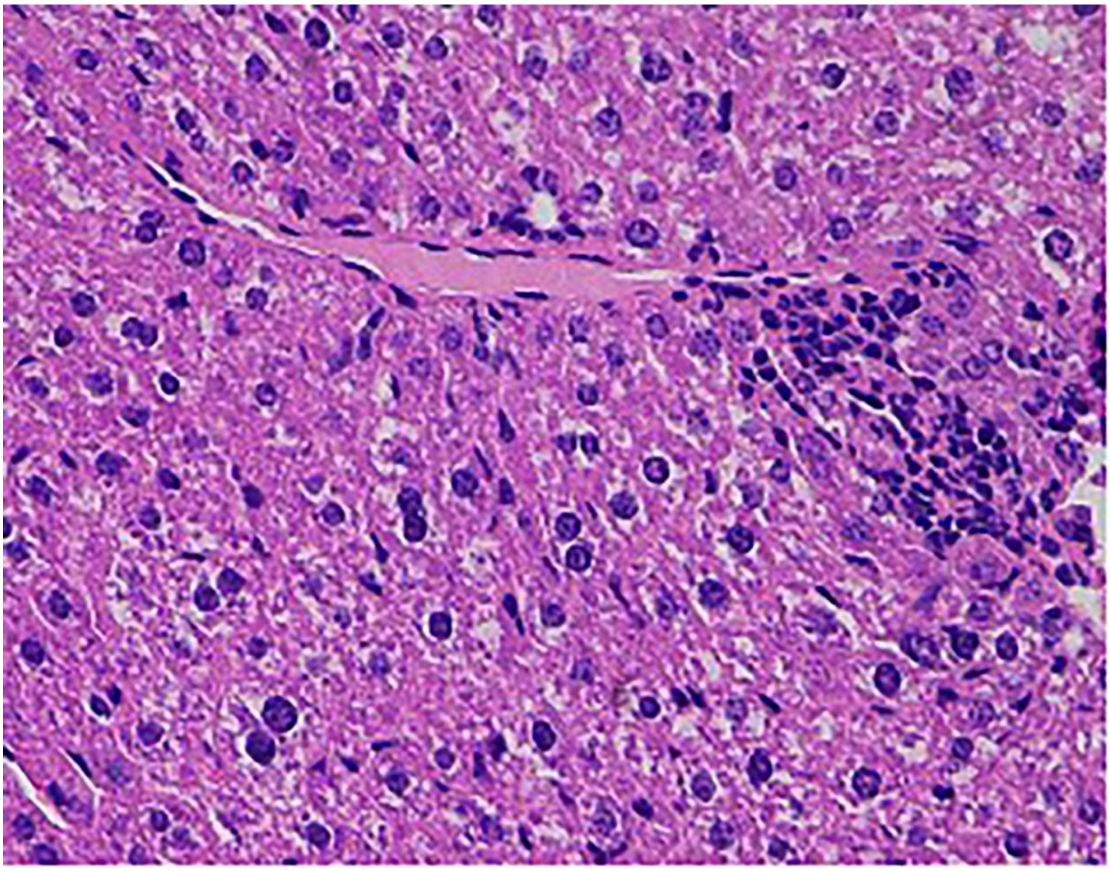

(E)
Spleen

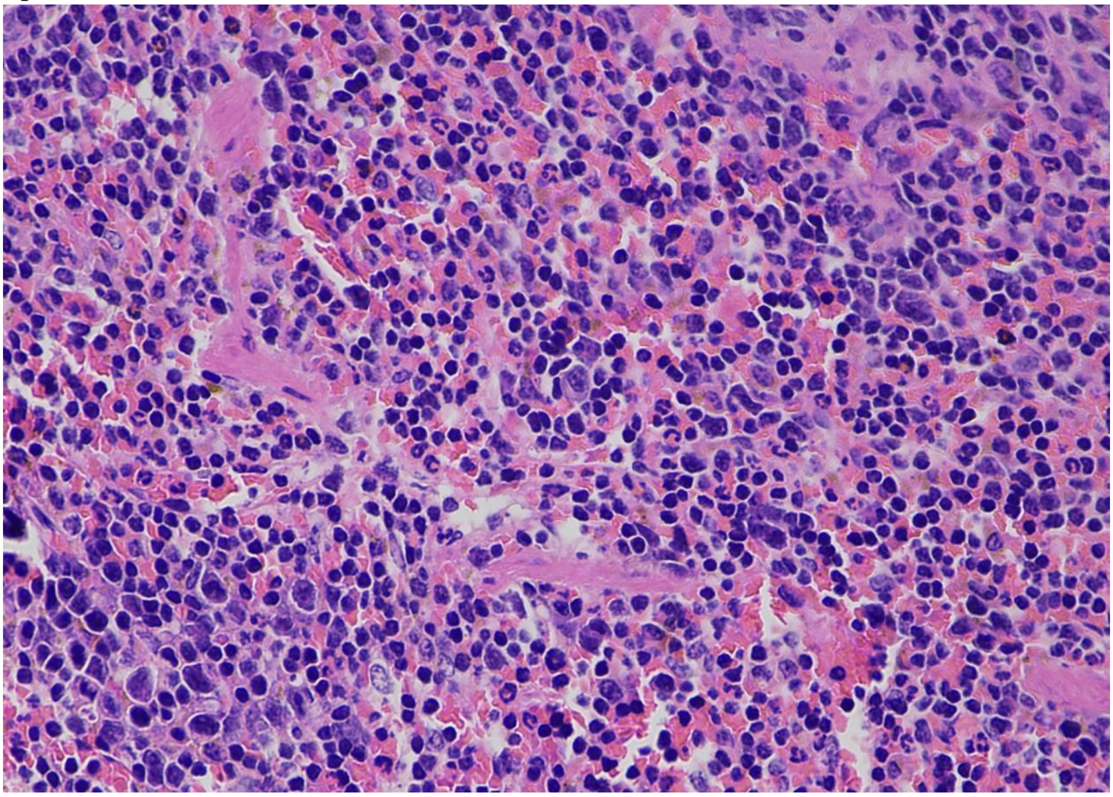

(F)
Cecum

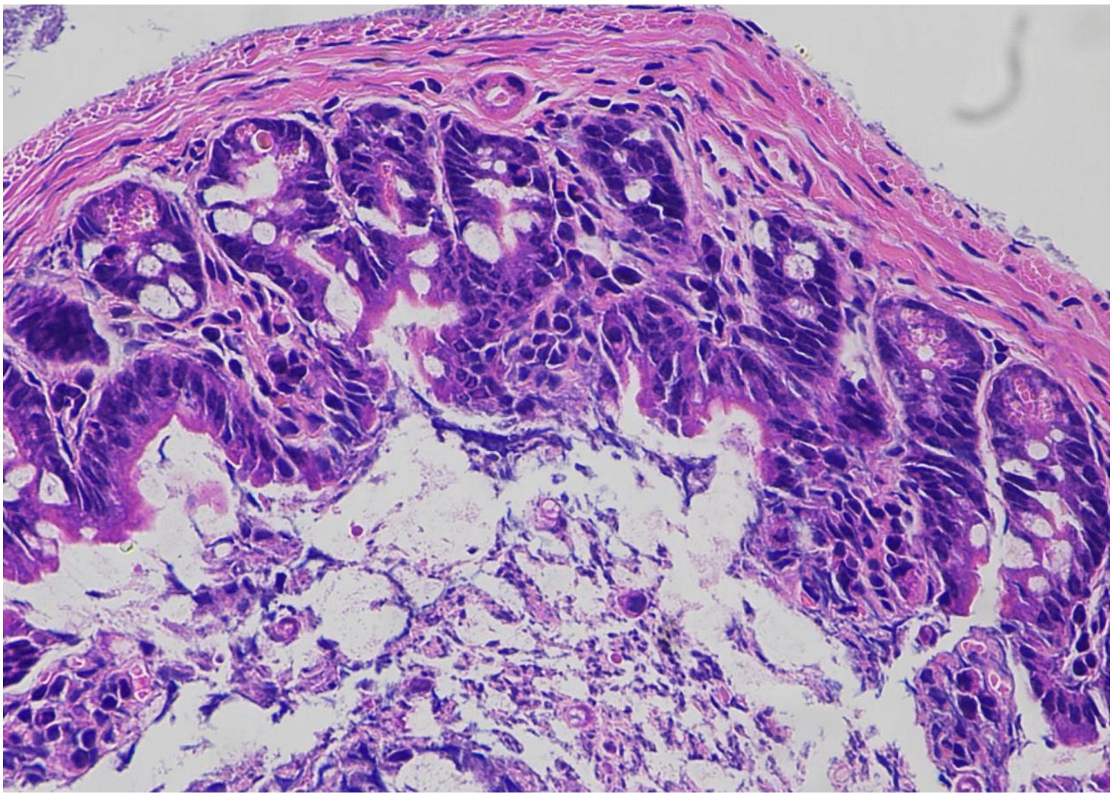
(G)
Liver
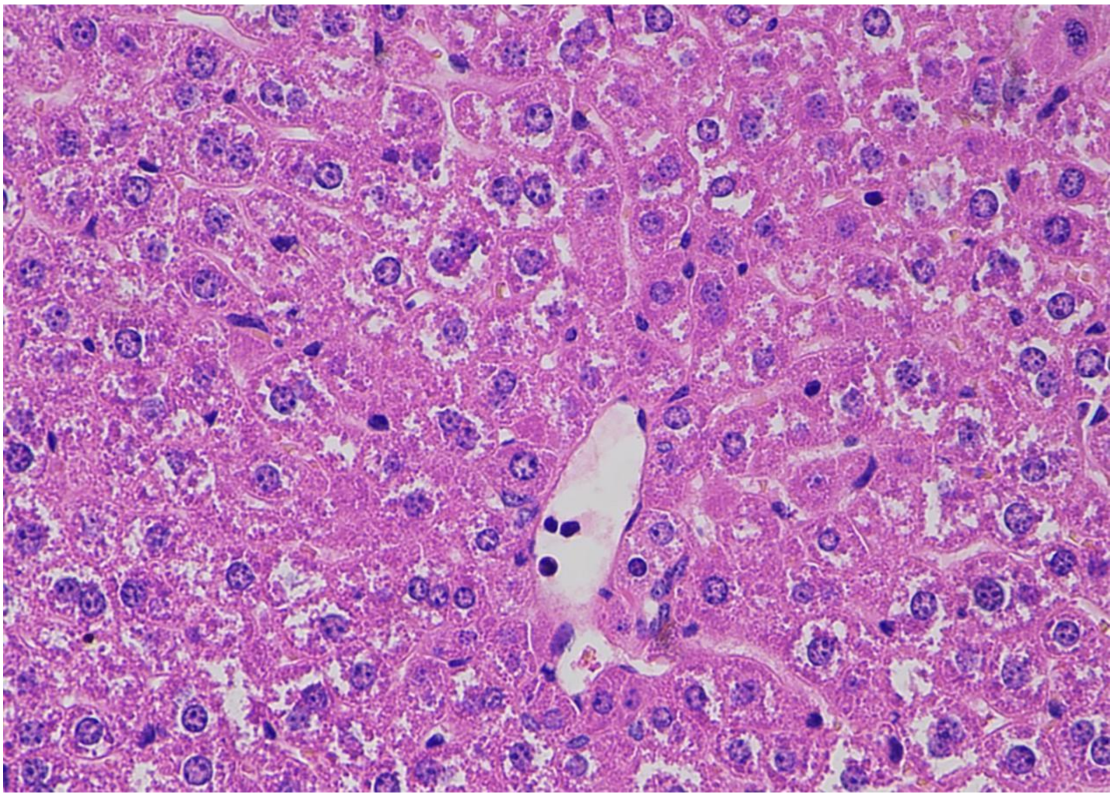
(H)
Spleen

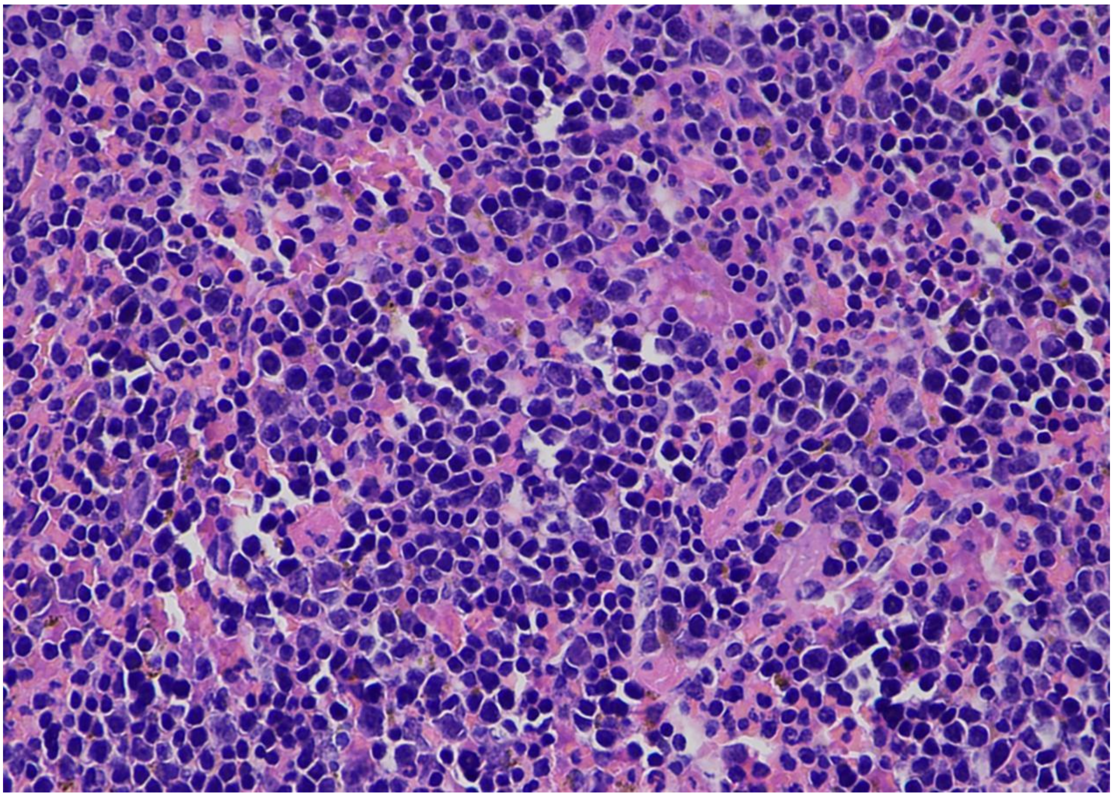
(I)
Cecum
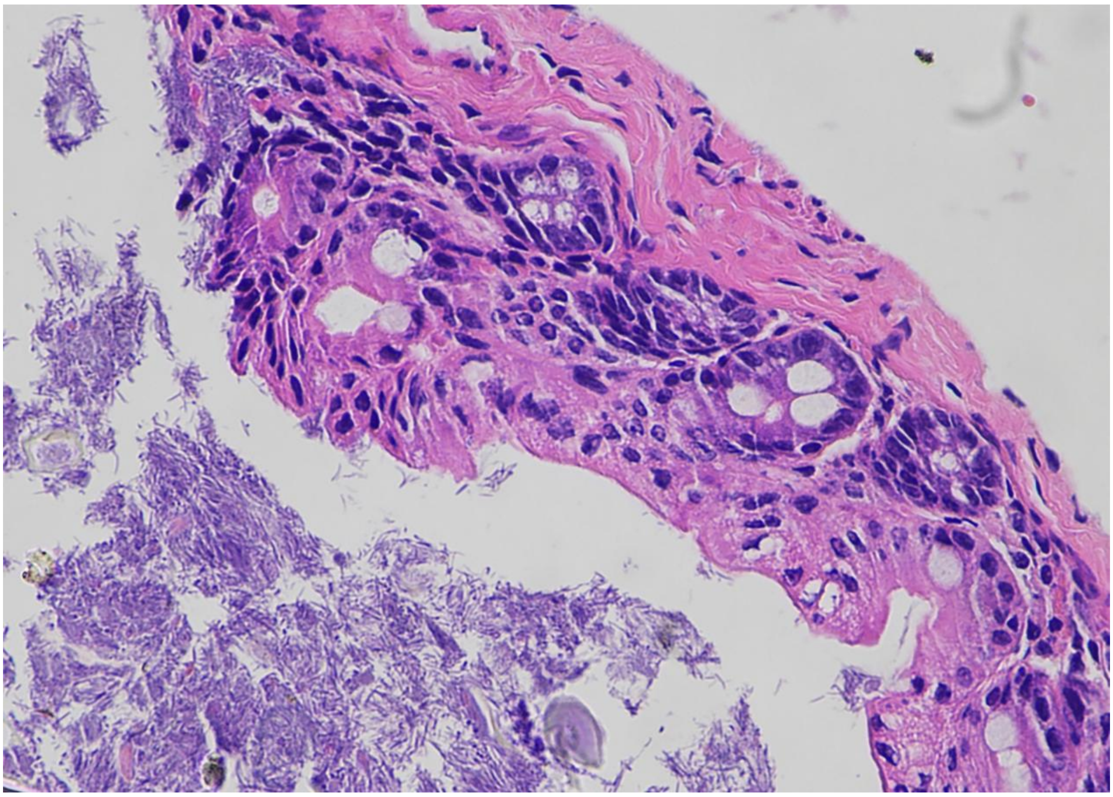
(J)
Liver

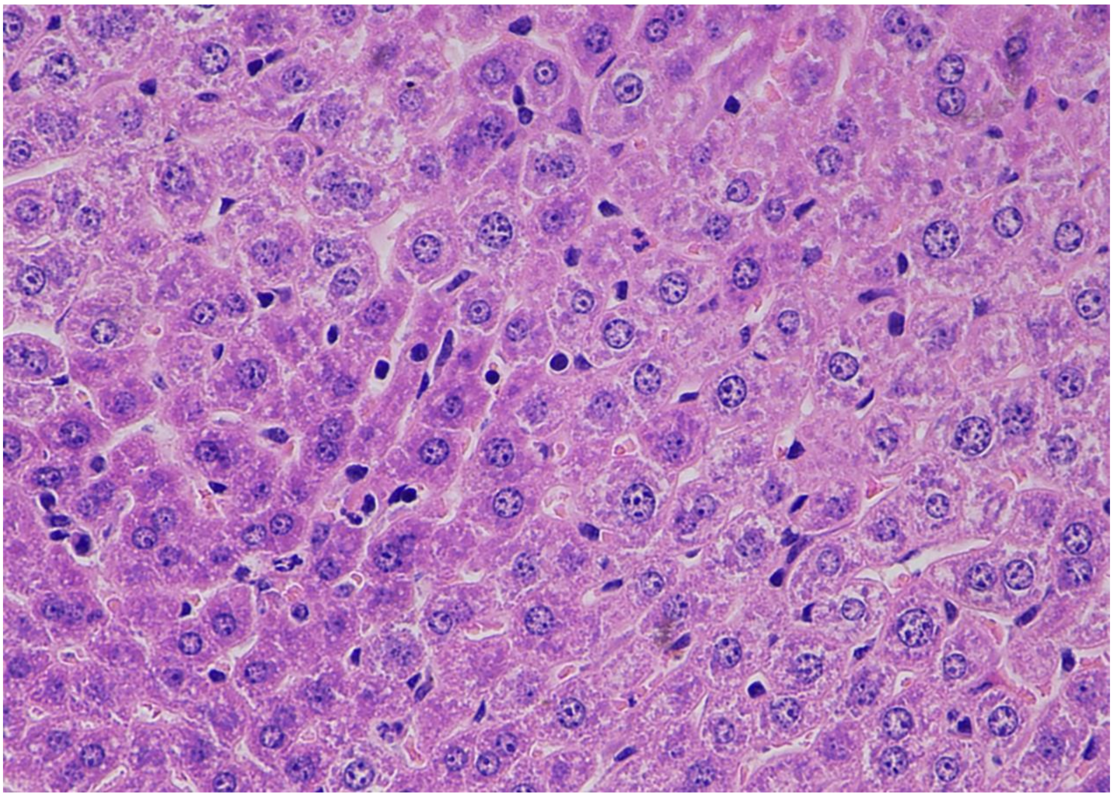

(K)
Spleen

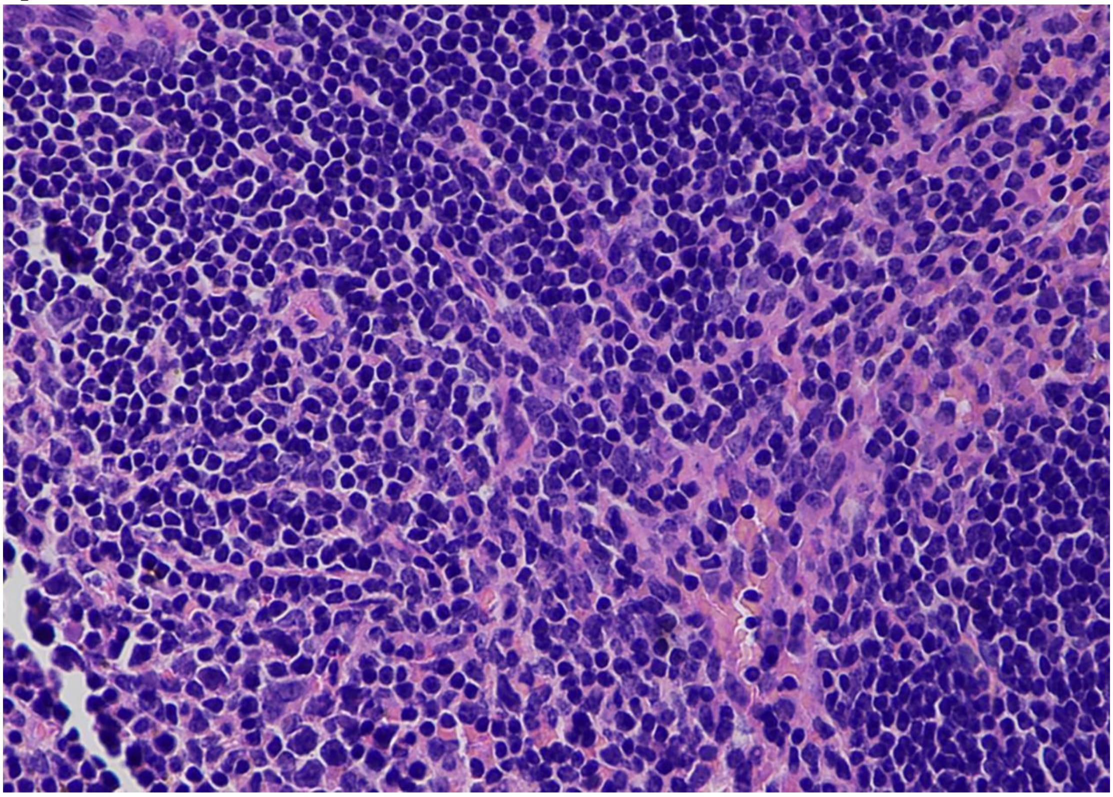

(L)
Cecum

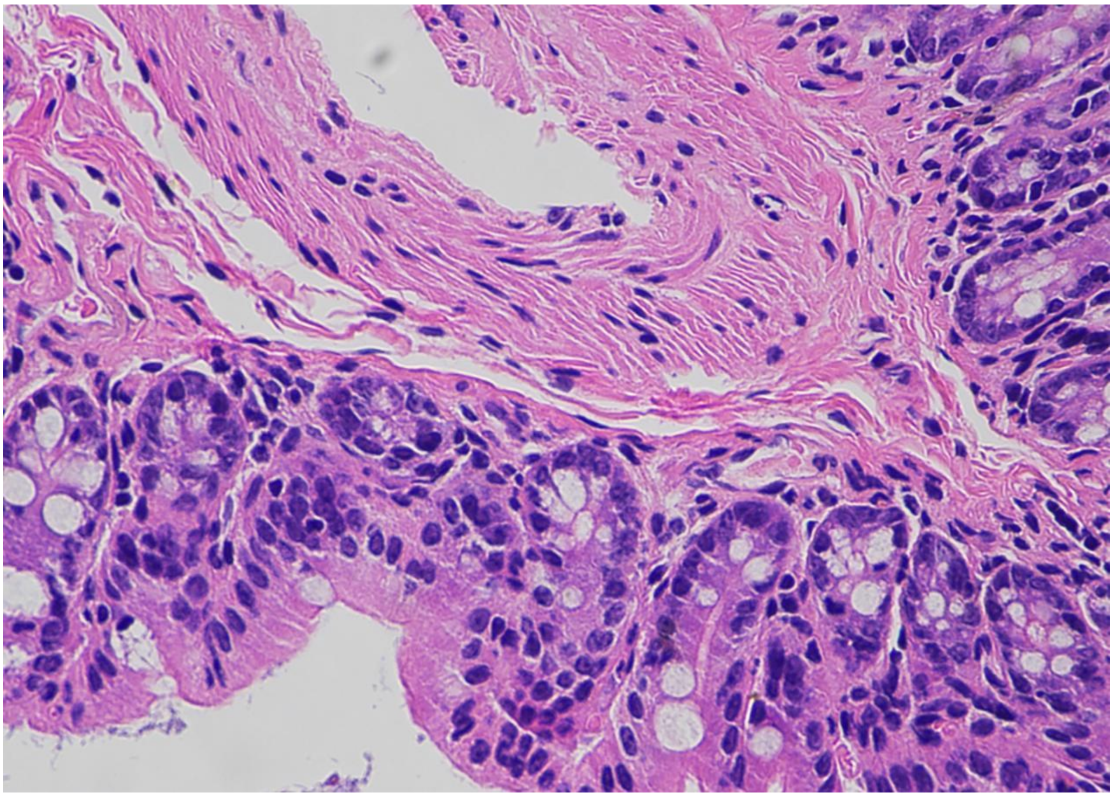

**Supplementary Figure 1.** Histological analysis after immunization. Representative images of haematoxylin and eosin-stained spleen, liver, and cecum tissue sections at 3 d post i.m. injection. (A), (B), and (C) are pathological sections of PBS group. (D), (E), and (F) are pathological sections of Sdu189 group. (G), (H), and (I) are pathological sections of Sdu189Δ*spiC* group. (J), (K), and (L) are pathological sections of Sdu189Δ*spiC*Δ*aroA* group (magnification is 400×).

(A)
Liver

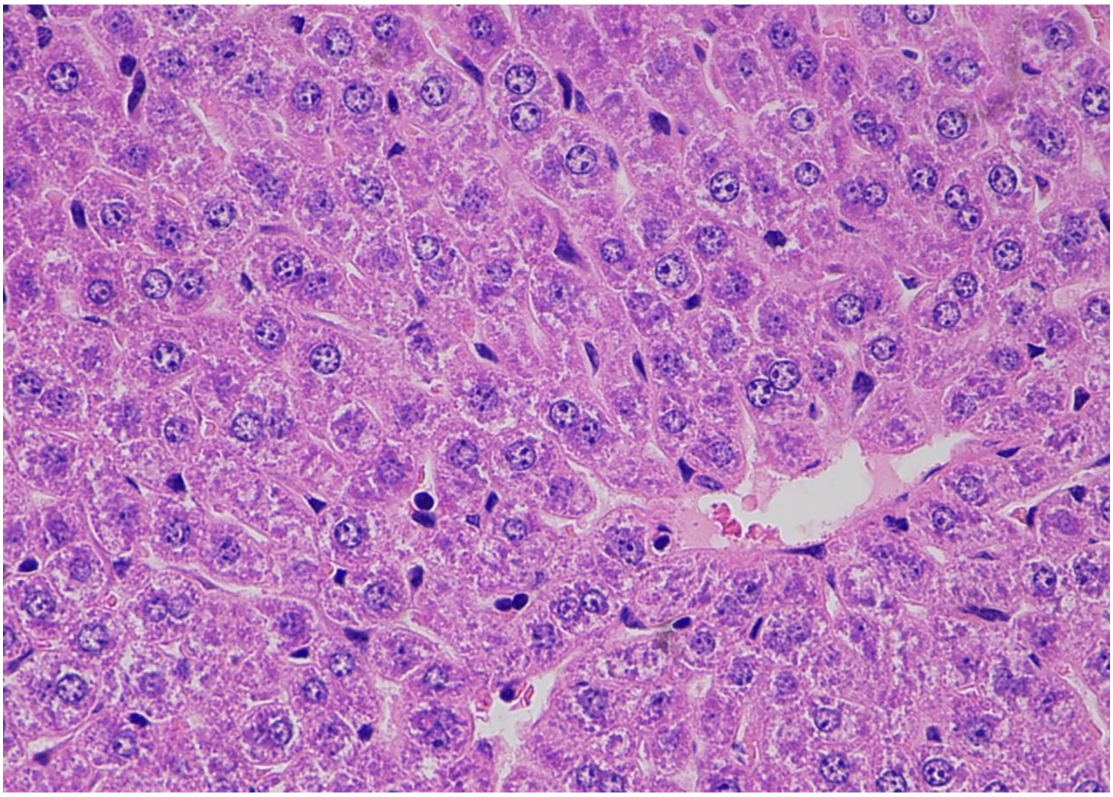

(B)
Spleen

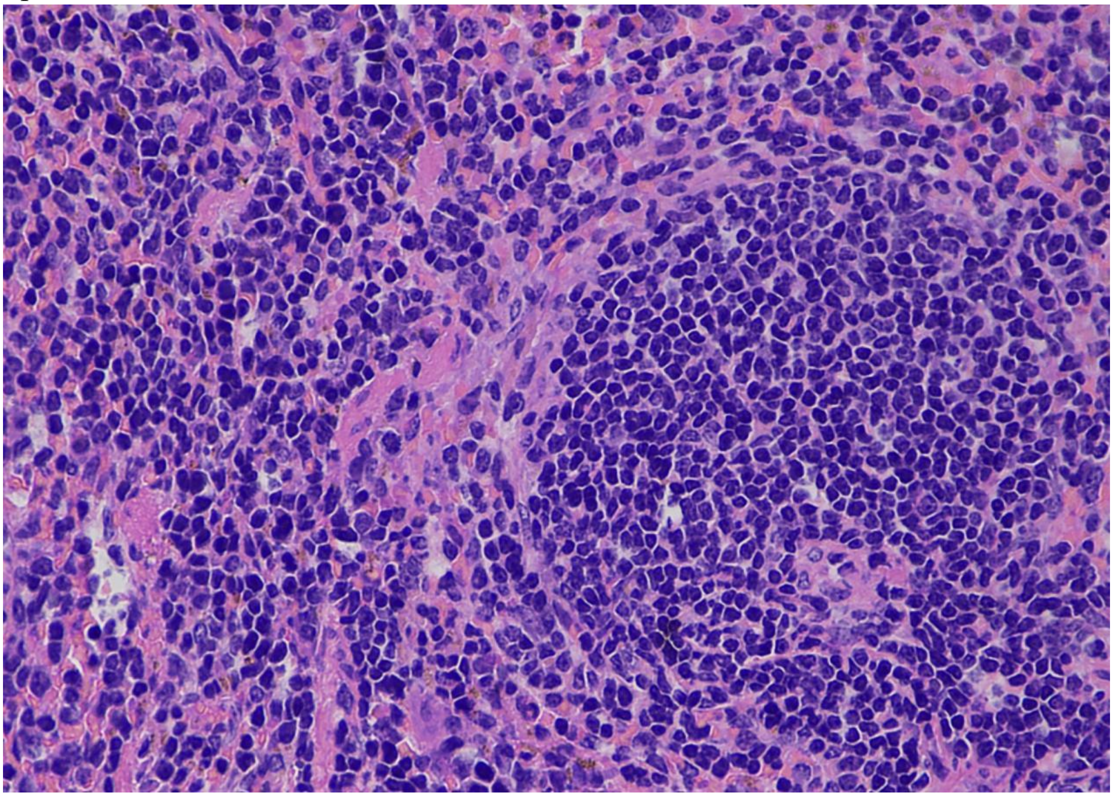

(C)
Cecum

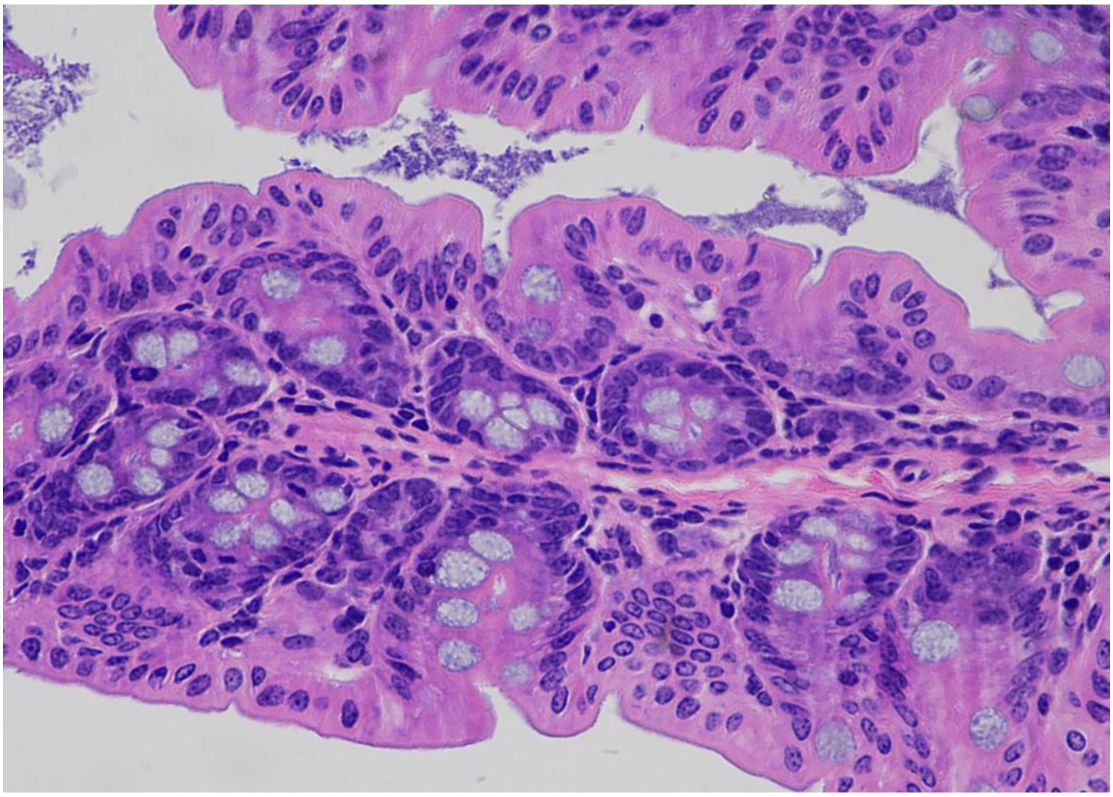

(D)

Liver

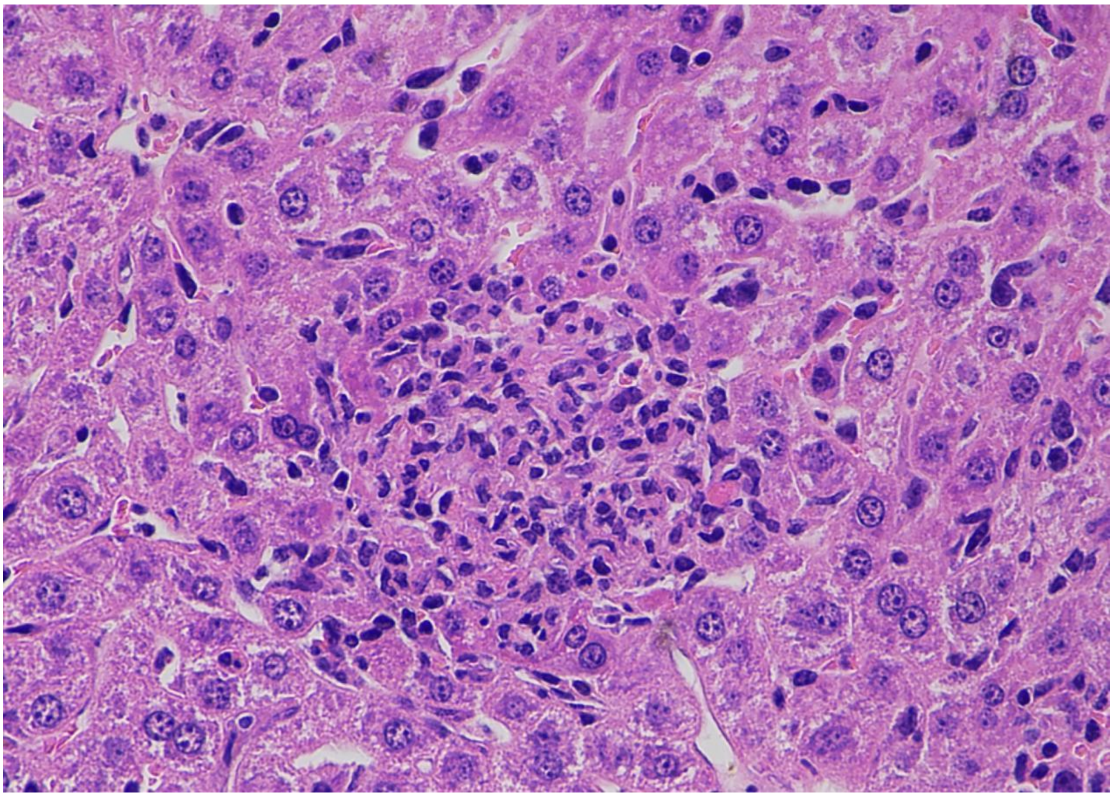

(E)

Spleen

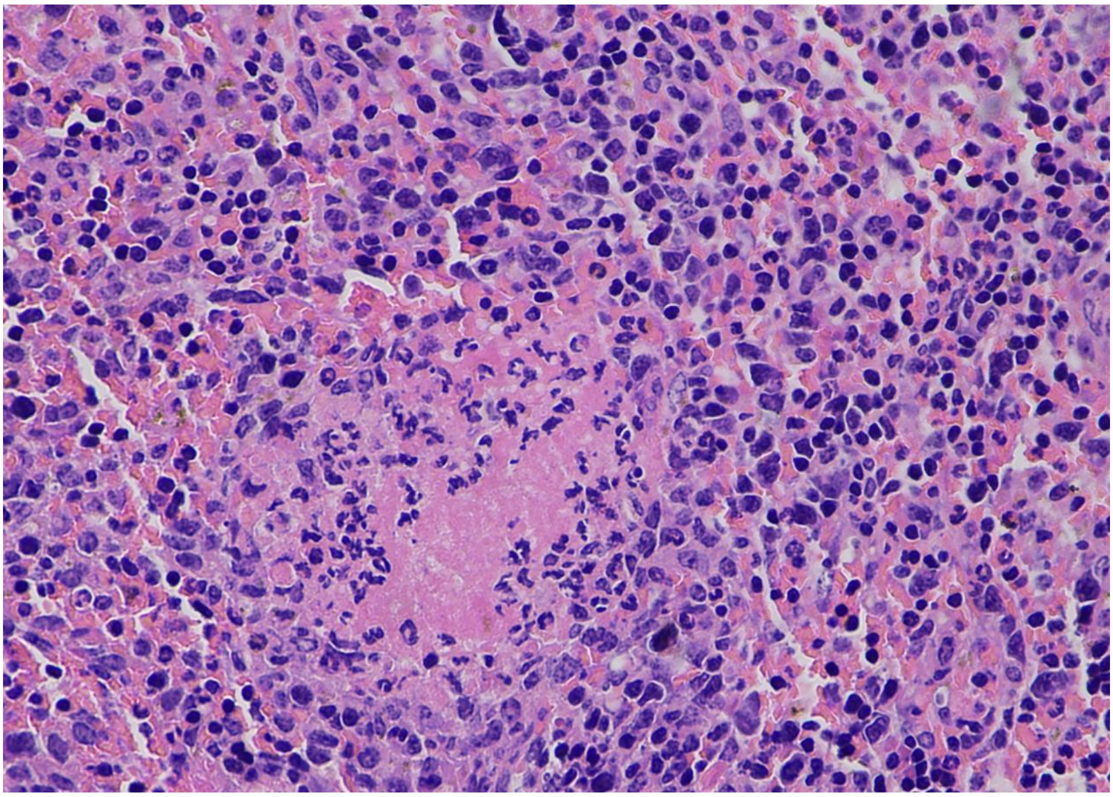

(F)
Cecum

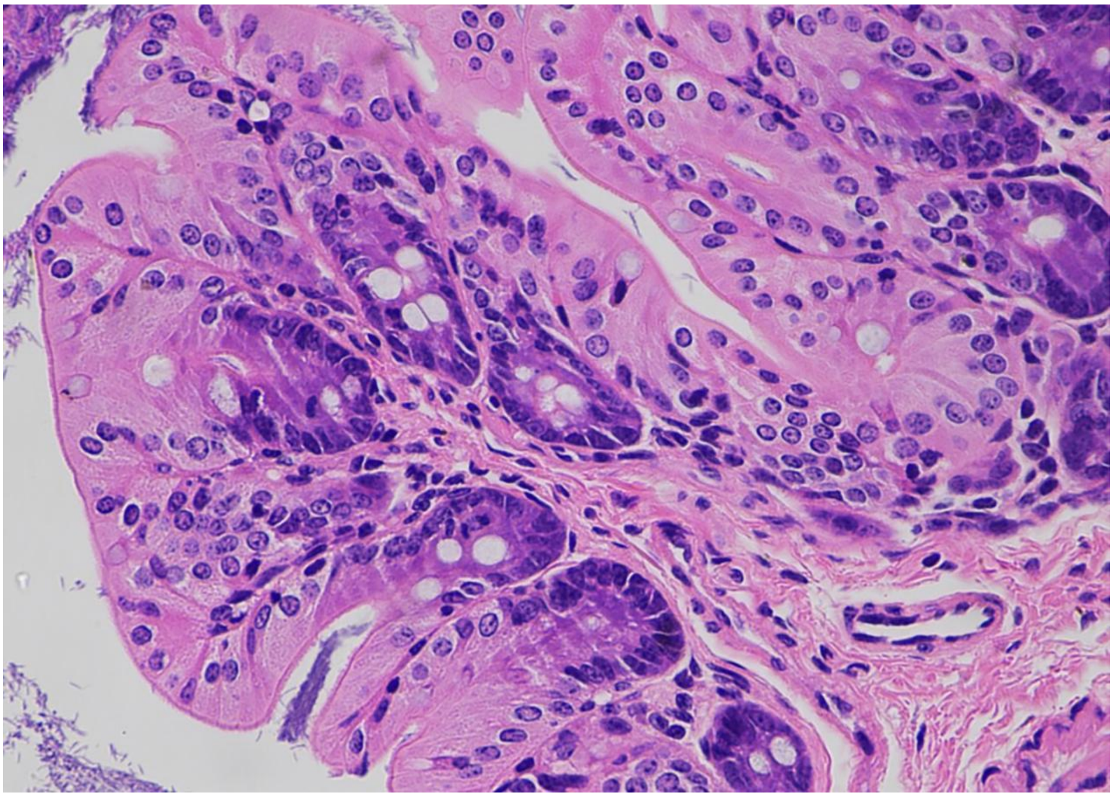

(G)
Liver

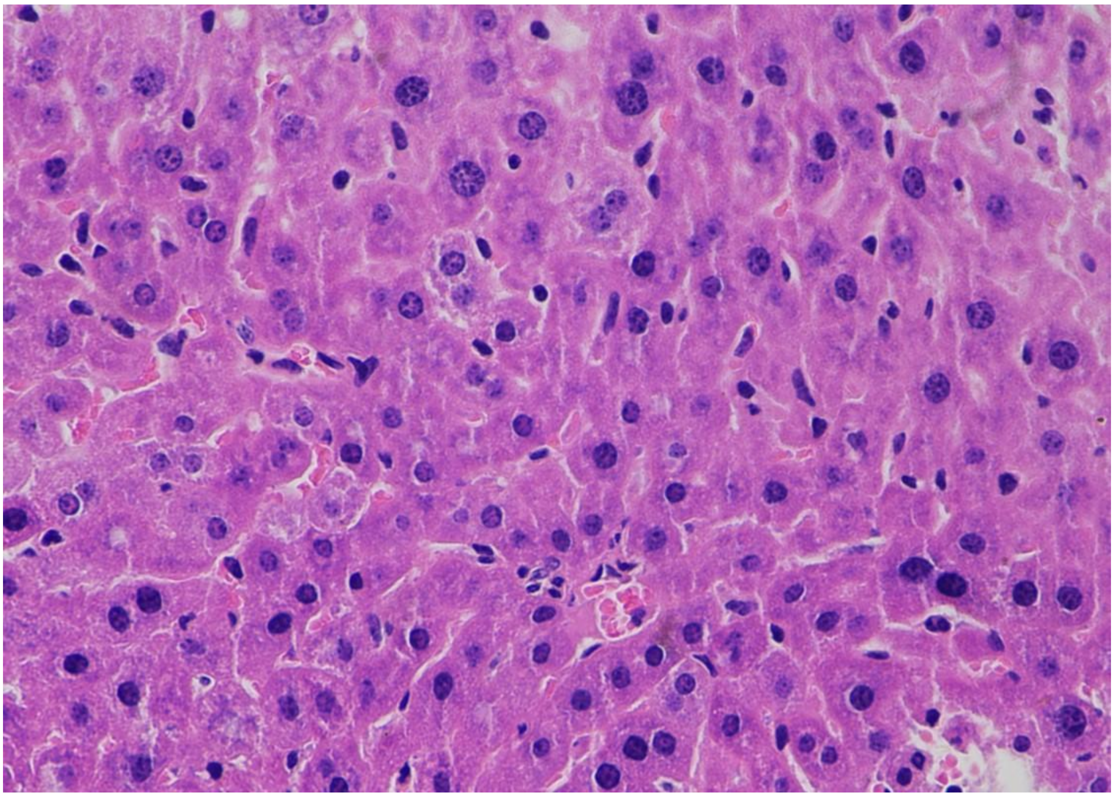

(H)

Spleen

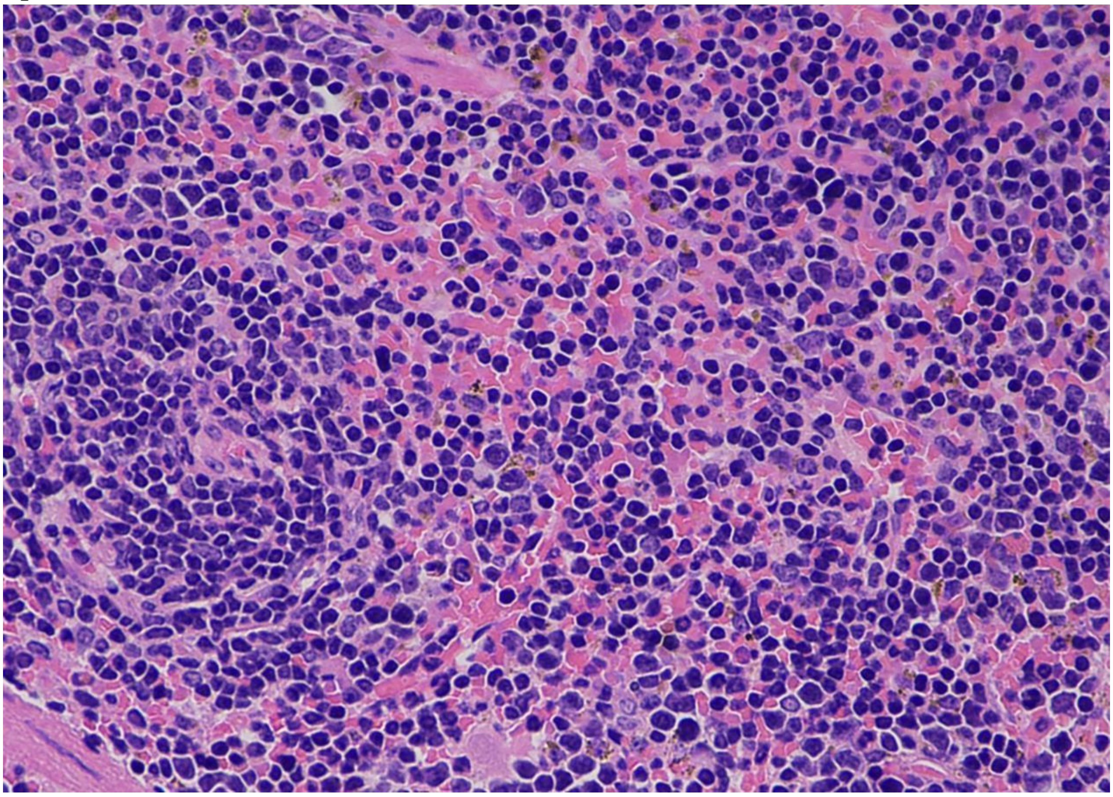

(I)

Cecum

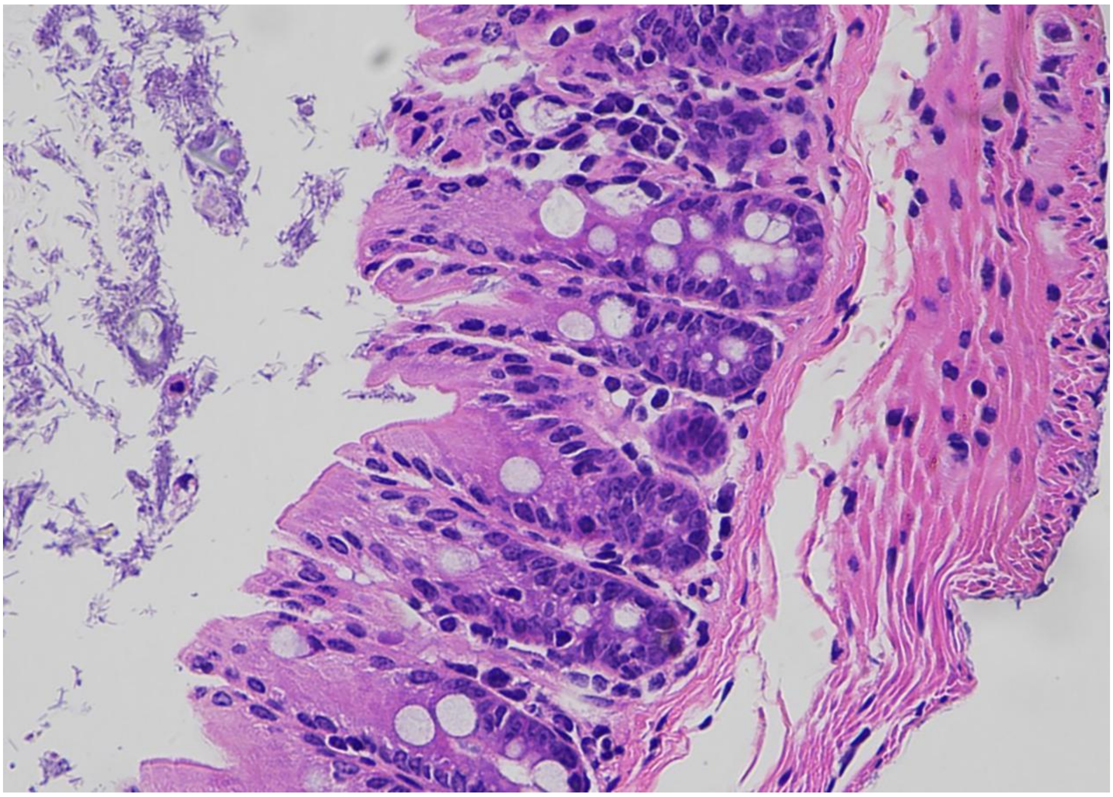

(J)
Liver

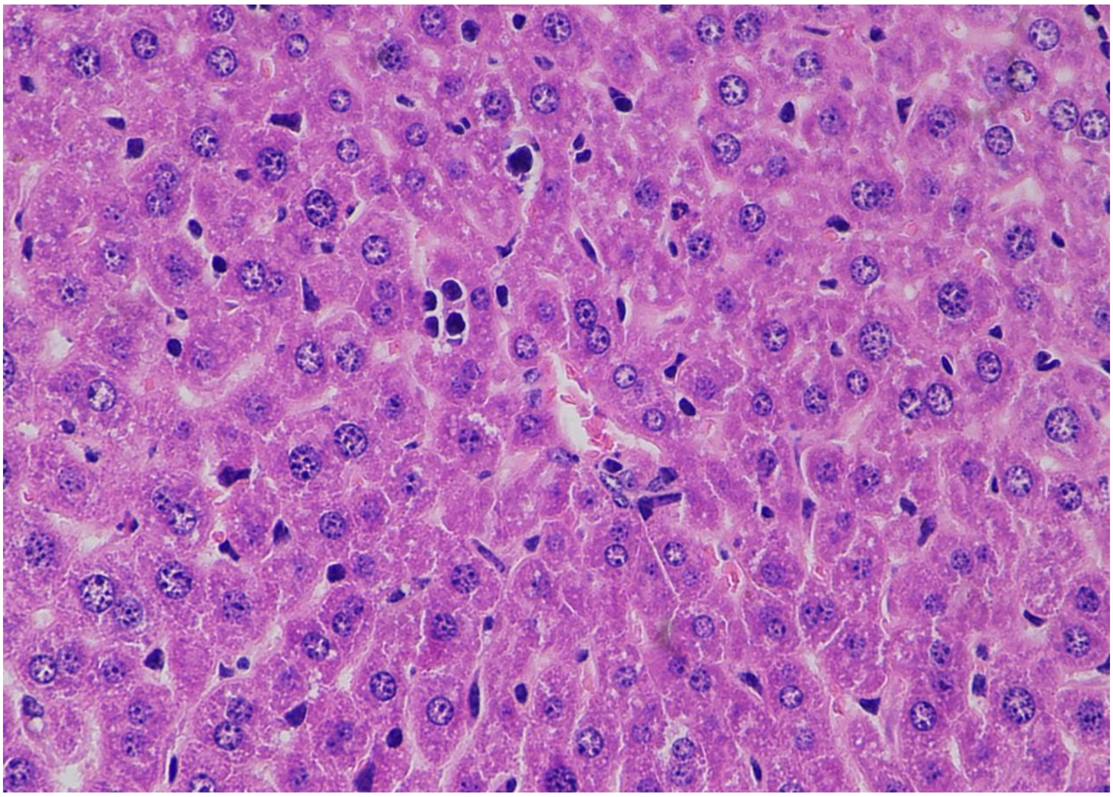

(K)
Spleen

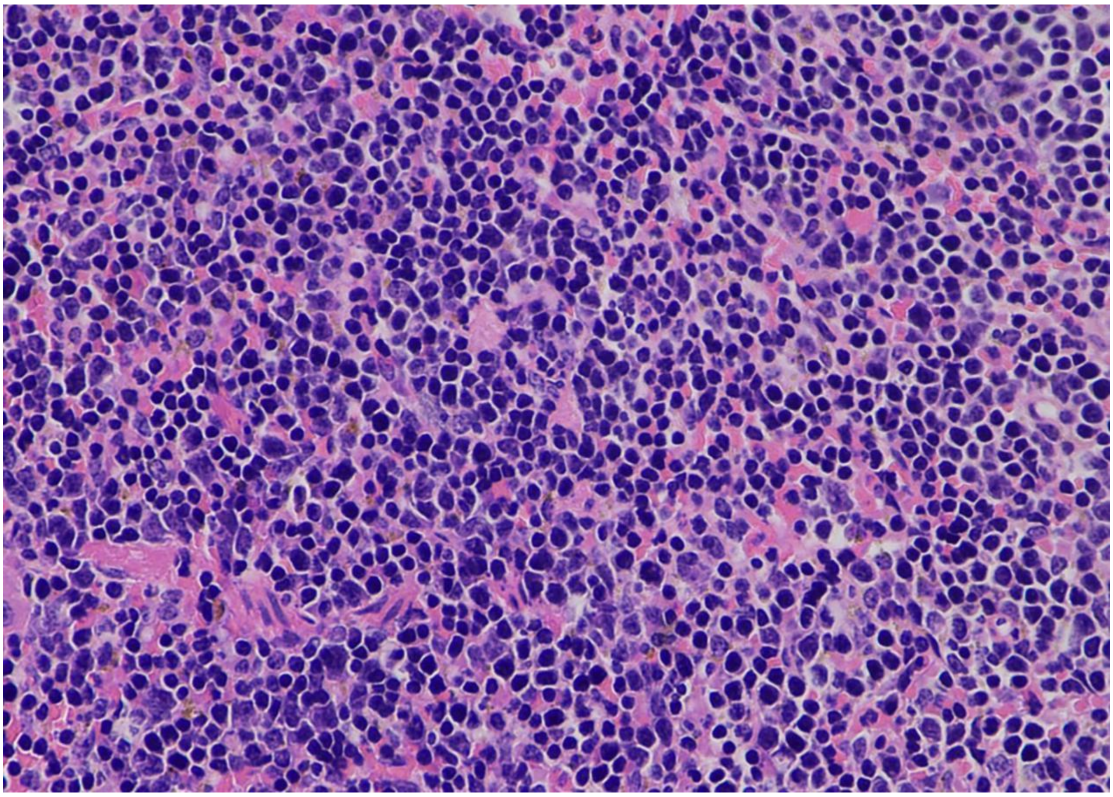

(L)

Cecum

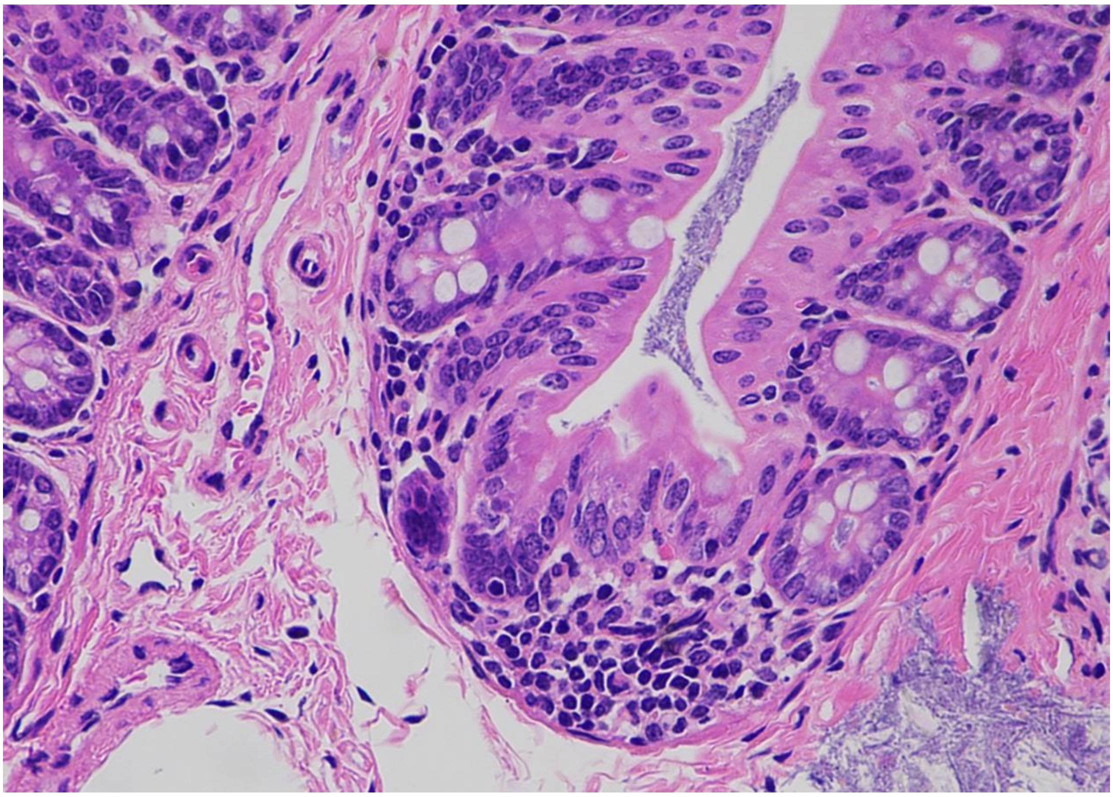

**Supplementary Figure 2.** Histological analysis after immunization. Representative images of haematoxylin and eosin-stained spleen, liver, and cecum tissue sections at 14 d post i.m. injection. (A), (B), and (C) are pathological sections of PBS group. (D),

(E), and (F) are pathological sections of Sdu189 group. (G), (H), and (I) are pathological sections of Sdu189Δ*spiC* group. (J), (K), and (L) are pathological sections of Sdu189Δ*spiC*Δ*aroA* group (magnification is 400×).
